# Corrigendum to “Performance of an open machine learning model to classify sleep/wake from actigraphy across ~24-hour intervals without knowledge of rest timing” [Sleep Health 9 (2023) 596–610]

**DOI:** 10.1016/j.sleh.2023.11.008

**Published:** 2024-02-15

**Authors:** Daniel M. Roberts, Margeaux M. Schade, Lindsay Master, Vasant G. Honavar, Nicole G. Nahmod, Anne-Marie Chang, Daniel Gartenberg, Orfeu M. Buxton

**Affiliations:** a Department of Biobehavioral Health, The Pennsylvania State University, University Park, Pennsylvania, USA; b Proactive Life, Inc, New York, New York, USA; c Faculty of Data Sciences, College of Information Science and Technology, The Pennsylvania State University, University Park, Pennsylvania, USA

The authors regret a labeling error affecting 12 of the 220 days of data used within the main analysis: 10 days within the “Deep Sleeping” dataset, and 2 days within the “EcoSleep” dataset. Specifically, a daylight savings time change that occurred following actigraphy data collection start but before participation in the staged days led to incorrect time sync between the actigraphy and sleep staging on those 12 days. Although a minority of records, this mislabeling of ground truth likely impaired model training, and also decreased the reported performance for all classification types reported in the original manuscript, which all referenced the same ground truth labels. This error has been corrected in the current manuscript. In comparison to the earlier proof, the overall conclusions remain, though some statistical comparisons between the classifiers on the various metrics have crossed the .05 significance threshold, either becoming statistically significant, or no longer reaching statistical significance. Specifically, within the main manuscript, NPV for the ~24-hour interval no longer reaches significance, while PPV for the in-bed interval does now reach significance (Table 6). Bias in SE for the in-bed interval no longer reaches significance, while MAE in SOL and CCC in SE for the in-bed interval do now reach significance (Table 7). Similarly, some statistical tests within the Supplementary Material have crossed the .05 threshold in either direction, though the overall conclusions remain the same. The authors would like to apologize for any inconvenience caused.

## Methods

For the computation of the updated results within the corrigendum, the Keras Tuner package was updated from version 1.1.0 to 1.1.3.

## Results

### Core analytics and main outcome variables

#### Epoch-by-epoch performance

Epoch-by-epoch performance is indicated within Table 6. At the ~24-hour interval, the TCN model produces favorable epoch-by-epoch performance to the Oakley classifications on nearly all the metrics evaluated, excepting sensitivity and NPV which do not statistically differ. When restricting the performance evaluation to only the known in-bed interval, the TCN shows favorable epoch-by-epoch performance on accuracy, PPV, NPV, F1-score, Matthews correlation coefficient, and PABAK, while the remaining measures do not statistically differ between the two classifiers.

Fig. 3 displays ROC curves for the TCN classifier, separately for ~24-hour and in-bed evaluation. The ROC curve depicts the trade-off between the true positive rate (sensitivity) and the false positive rate (1 – specificity) as the probability threshold for classification is altered. The model performs more favorably across the ~24-hour interval than the in-bed interval, also reflected numerically by the AUC values in Table 6.

Table 7 displays confusion matrices for the performance of the Oakley and TCN classifiers, at both evaluation intervals. The confusion matrices reiterate the increased specificity for the TCN model at ~24-hour evaluation that had been demonstrated statistically in Table 6. In addition, by summing within columns, the base rates of sleep and wake within each interval can be obtained.

### Sleep metric discrepancy performance at ~24-hour and in-bed intervals

Discrepancy of classifiers within the ~24-hour and in-bed intervals are indicated in Table 8 for bias and MAE, and Table 9 for CCC/CCCLON. Across the ~24-hour interval, relative to the Oakley classifications, the amount of TST predicted by the TCN model more closely matches ground truth in terms of bias, MAE, and both variants of CCC. Scatterplots and Bland-Altman plots visualizing the relationship between true and predicted TST across the ~24-hour interval are shown in Fig. 4. Within the in-bed interval, the classifiers have fewer differences in terms of reproducing ground truth. The Oakley classifier performs significantly better in terms of bias in SE or WASO, while the TCN classifier performs significantly better in terms of bias, MAE, and both variations of CCC in SOL, and CCC in SE. Scatterplots and Bland-Altman plots visualizing the relationship between true and predicted values for these metrics across the in-bed interval are shown in Fig. 5.

## Supplementary Material

1

Appendix A. Supporting information

Supplementary data associated with this article can be found in the online version at doi:10.1016/j.sleh.2023.11.008.

## Figures and Tables

**Fig. 3.**
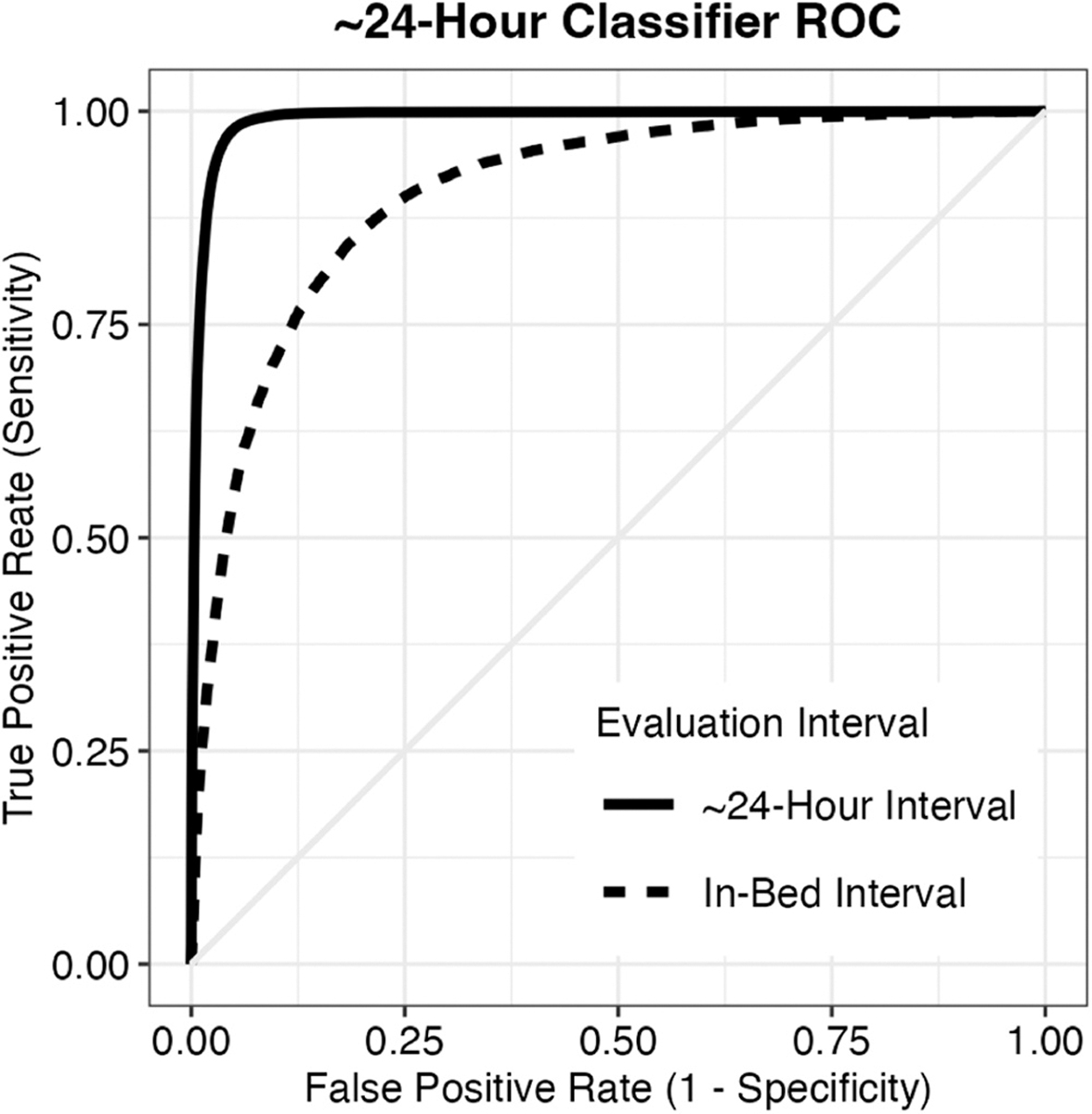
Receiver operating characteristic (ROC) for classifier performance, collected across the 5 cross-validation folds. Performance is separately displayed for evaluation across the ~24-hour interval (solid line) or only within the in-bed interval (dashed line). Each line is the mean of ROC curves from individual days, combined via threshold averaging. These curves do not account for clustering of days within participants, however mean area under the ROC curve values derived from mixed-effects models accounting for clustering are displayed in Table 6.

**Fig. 4.**
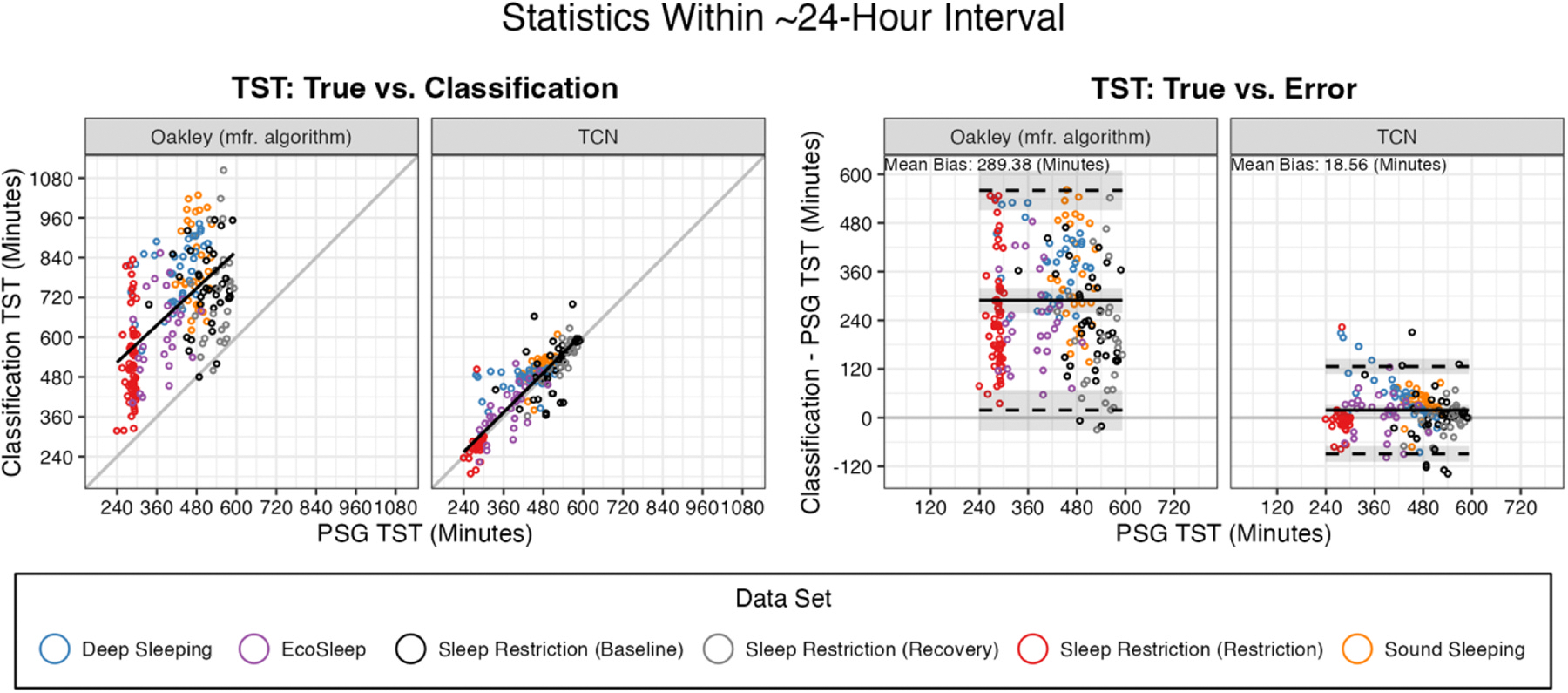
Polysomnography (PSG)-derived total sleep time (TST) over the ~24-hour interval, compared to the Oakley or temporal convolutional network classifiers. Left column shows PSG vs. classification for each classification type, and for visual reference includes a linear regression of predicted TST on true TST across days (without accounting for clustering within participants; black line). Right column shows Bland-Altman PSG vs. classification error (classification TST - PSG TST), computed using a mixed-effects approach accounting for clustering of days within participants. Proportional bias was evaluated but did not reach statistical significance for either classifier on ~24-hour TST. Indicated are bias (black line) and the upper and lower limits of the 95% limits of agreement (dashed black lines) with 95% confidence intervals for each shaded in gray.

**Fig. 5.**
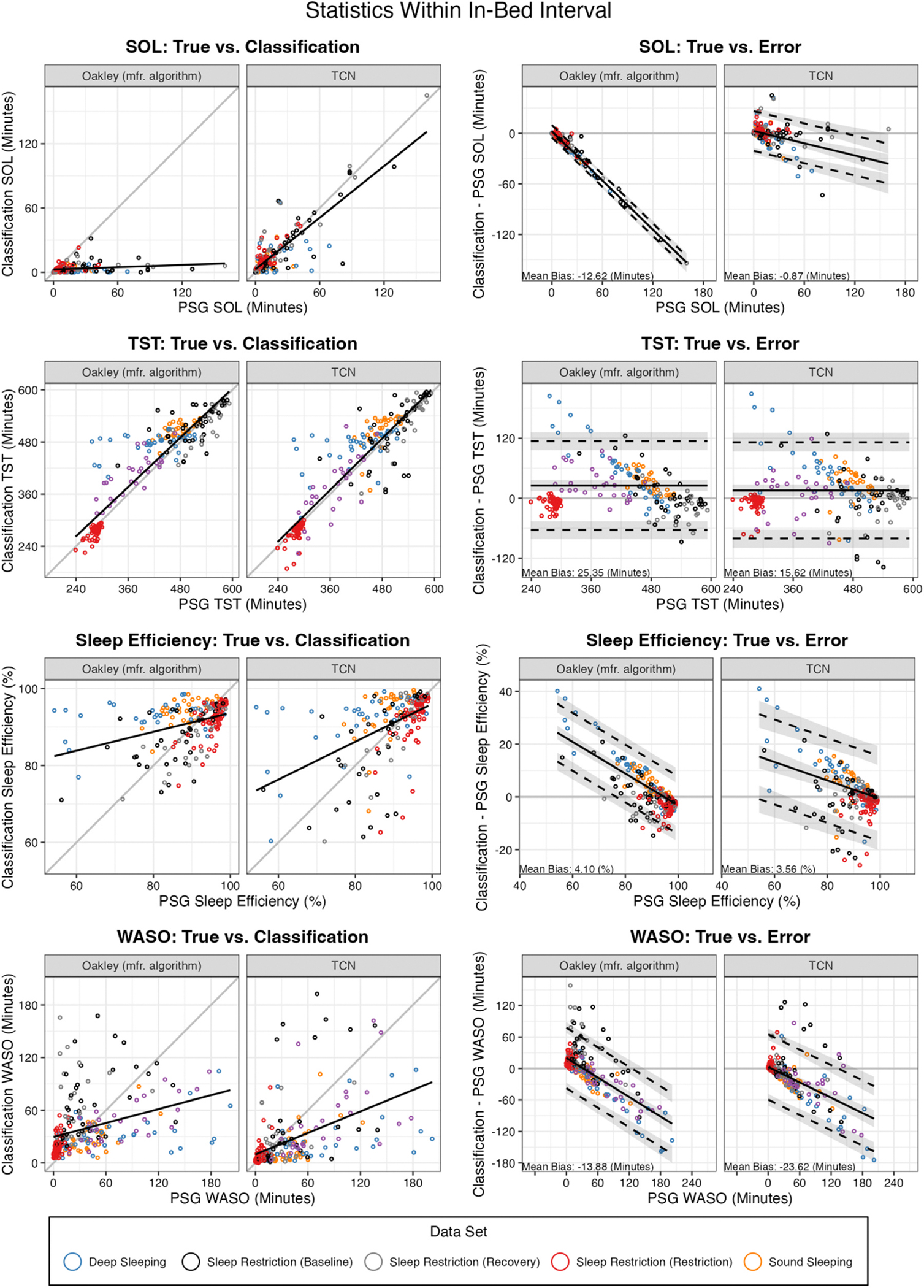
Polysomnography (PSG)-derived sleep metrics restricted to the in-bed interval (as defined by registered polysomnography technicians or algorithm in the case of EcoSleep), compared to values from the Spectrum classification outputs or our temporal convolutional network classifier restricted to the same interval. Left column shows the scatterplot of PSG vs. classification derived metric for each classification type, and for visual reference includes the linear regression of predicted metric on true metric (without accounting for clustering within participants; black line). Right column shows a Bland-Altman plot of PSG metric vs. classification metric error (classification metric - PSG metric), computed using a mixed-effects approach accounting for clustering of days within participants. Plotted are sleep onset latency (SOL), total sleep time (TST), sleep efficiency (SE), and wake after sleep onset (WASO). Proportional bias was evaluated and reached significance for all statistics except for TST, which did not reach significance for either classifier. Indicated are bias (black line) and the upper and lower limits of the 95% limits of agreement (dashed black lines), with 95% confidence intervals for each shaded in gray. The EcoSleep dataset was excluded from comparisons of SOL or SE, as the method of imputing in-bed labels for the EcoSleep data cannot identify presleep wakefulness within the in-bed interval.

**Table 3:** Data remaining following recording-level rejection. Standard deviation is indicated in parentheses.

Data set	Mean age (SD age)	Gender	Percent of epochs without staging	Percent of epochs within staging without actigraphy counts	Percent of epochs prestaging without actigraphy counts	Percent of epochs poststaging without actigraphy counts	Percent of recordings that could not have actigraphy alignment applied	Average actigraphy lag identified (s)	Percent of records with lag of 0 epochs (no adjustment needed)	Valid epochs per recording	Valid epochs per recording within in-bed interval

Deep sleeping	50.9 (4.0)	4 M/8 F	0.4 (0.2)	1.8 (2.8)	10.4 (26.6)	34.4 (37.3)	0	− 35.0 (225.7)	13.9	2813.4 (79.9)	1024.2 (14.9)
EcoSleep	77.7 (5.2)	5 M/12 F	5.0 (4.7)	2.4 (3.7)	41.0 (42.9)	3.5 (6.0)	0	− 284.4 (290.7)	29.6	2418.8 (158.3)	898.9 (116.4)
MESA	69.3 (9.1)	773 M/917 F	0.0 (0.0)	0.4 (1.9)	46.8 (32.4)	4.2 (13.6)	0	89.0 (171.9)	49.2	1060.2 (178.1)	951.3 (163.0)
Sleep restriction	22.3 (2.8)	15 M/0 F	0.9 (1.8)	2.8 (3.9)	6.4 (14.4)	13.7 (16.3)	2.4	− 16.8 (44.5)	63.5	2771.1 (119.4)	892.1 (298.0)
Sound sleeping	40.2 (5.0)	3 M/5 F	0.2 (0.2)	2.9 (4.4)	7.8 (9.2)	14.6 (24.3)	0	− 41.6 (34.4)	22.6	2789.1 (126.1)	1078.2 (16.4)

MESA, Multi-Ethnic Study of Atherosclerosis.

**Table 5:** Optimal hyperparameters identified within each fold of the model

Hyperparameter	Fold 1	Fold 2	Fold 3	Fold 4	Fold 5

Number of filters in TCN	64	64	64	64	64
TCN kernel size	7	7	7	7	7
Set of TCN dilations	[1, 2, 4, 8, 16, 32]	[1, 2, 4, 8, 16, 32]	[1, 2, 4, 8, 16, 32]	[1, 2, 4, 8, 16, 32]	[1, 2, 4, 8, 16, 32]
Dropout inside TCN	0	0	0.2	0	0
Normalization	Layer normalization	Layer normalization	None	None	None
Dropout prior to dense layer	0	0.5	0	0.3	0.3
Learning rate	2.27E-04	1.08E-04	3.53E-04	5.74E-04	1.65E-04

TCN, temporal convolutional network.

**Table 6:** Epoch-level classification performance for both the ~24-hour and in-bed intervals for both the Oakley and TCN classifiers

Metric	Oakley (Grand)	TCN (Grand)	Oakley (Mixed)	TCN (Mixed)	Difference	DF	t	*p*	d	Random effect structure

*~24-hour interval epoch-by-epoch evaluation*										
AUC		0.993 (0.011)		0.989 (0.012; 0.007)						
Accuracy	0.774 (0.089)	0.963 (0.035)	0.762 (0.062; 0.065)	**0.955 (0.026; 0.026)**	0.201	47.17	21.03	< .01	3.33	Slope and intercept
Balanced accuracy	0.824 (0.063)	0.960 (0.038)	0.817 (0.040; 0.050)	**0.952 (0.032; 0.028)**	0.140	42.35	17.66	< .01	2.79	Slope and intercept
Sensitivity	0.954 (0.036)	0.954 (0.069)	0.959 (0.029; 0.018)	0.946 (0.062; 0.047)	− 0.012	36.35	− 1.42	.163	0.17	Slope and intercept
Specificity	0.693 (0.134)	0.967 (0.041)	0.674 (0.093; 0.099)	**0.958 (0.029; 0.032)**	0.273	392.70	33.79	< .01	2.75	Intercept
PPV	0.587 (0.125)	0.934 (0.082)	0.578 (0.073; 0.102)	**0.912 (0.067; 0.055)**	0.345	41.68	29.41	< .01	3.33	Slope and intercept
NPV	0.972 (0.025)	0.981 (0.029)	0.974 (0.014; 0.019)	0.977 (0.024; 0.021)	0.006	29.93	1.81	.081	0.19	Slope and intercept
F1-Score	0.719 (0.094)	0.940 (0.059)	0.713 (0.055; 0.078)	**0.925 (0.050; 0.041)**	0.221	41.37	22.68	< .01	2.87	Slope and intercept
MCC	0.600 (0.116)	0.917 (0.076)	0.589 (0.075; 0.092)	**0.896 (0.063; 0.055)**	0.315	41.17	22.96	< .01	3.28	Slope and intercept
PABAK	0.548 (0.177)	0.927 (0.069)	0.525 (0.125; 0.131)	**0.910 (0.051; 0.052)**	0.402	47.17	21.03	< .01	3.33	Slope and intercept
*In-bed interval epoch-by-epoch evaluation*										
AUC		0.925 (0.071)		0.903 (0.062; 0.047)						
Accuracy	0.897 (0.070)	0.911 (0.080)	0.882 (0.060; 0.047)	**0.889 (0.071; 0.05l)**	0.011	48.32	2.16	.036	0.12	Slope and intercept
Balanced accuracy	0.744 (0.109)	0.773 (0.124)	0.718 (0.078; 0.076)	0.738 (0.091; 0.083)	0.020	50.95	1.89	.065	0.17	Slope and intercept
Sensitivity	0.954 (0.036)	0.956 (0.067)	0.960 (0.028; 0.018)	0.951 (0.056; 0.047)	− 0.008	35.47	− 0.97	.337	0.12	Slope and intercept
Specificity	0.534 (0.232)	0.589 (0.265)	0.475 (0.173 ; 0.150)	0.524 (0.197; 0.175)	0.045	46.14	1.98	.053	0.18	Slope and intercept
PPV	0.930 (0.081)	0.942 (0.073)	0.907 (0.072; 0.052)	**0.919 (0.069; 0.042)**	0.012	386.09	2.82	< .01	0.14	Intercept
NPV	0.549 (0.213)	0.679 (0.225)	0.597 (0.147; 0.152)	**0.695 (0.146; 0.172)**	0.111	48.92	4.62	< .01	0.51	Slope and intercept
F1-Score	0.939 (0.047)	0.946 (0.054)	0.930 (0.041; 0.032)	**0.931 (0.051; 0.035)**	0.007	384.49	2.08	.038	0.11	Intercept
MCC	0.460 (0.142)	0.554 (0.182)	0.450 (0.085; 0.115)	**0.515 (0.130; 0.126)**	0.070	52.73	3.75	< .01	0.41	Slope and intercept
PABAK	0.793 (0.140)	0.821 (0.160)	0.765 (0.121; 0.095)	**0.779 (0.143; 0.103)**	0.021	48.32	2.16	.036	0.12	Slope and intercept

AUC, area under the receiver operating characteristic curve; DF, mixed-effects model degrees of freedom; MCC, Matthews correlation coefficient; NPV, negative predictive value; PABAK, prevalence-adjusted and bias-adjusted kappa; PPV, positive predictive value; t, mixed-effects model t-value for the comparison between classifiers; TCN, temporal convolutional network.

Bolded values indicate the more favorable outcomes for comparisons that are statistically significant at an alpha level of .05.

AUC not possible for Oakley classifier given discrete classification.

Mixed-effects models compared the manufacturer’s standard algorithm versus TCN model classification, with days nested within participants.

Grand mean values are averaged across recordings regardless of clustering within participants. The standard deviation of these values is represented in parentheses. Mixed-effect mean values are derived by fitting mixed-effects models separately to each condition with a fixed-effect of intercept, and a random-effect of intercept grouped within participants. Standard deviation values are at the level of the cluster, and residual.

Difference is the condition difference estimated from the mixed-effects model used to compute inferential statistics. This value is similar but not necessarily the same as the difference between the mixed-effect condition means, which are estimated independently.

For EcoSleep Study, in-bed interval was not set for participants, so nighttime sleep interval imputed from PSG scored start/end of nighttime sleep epochs.

**Table 7:** Epoch-level confusion matrices for the ~24-hour and in-bed intervals for both the Oakley and TCN classifiers

*(a) Confusion matrices for the ~24-hour and in-bed intervals computed as “grand” statistics without accounting for clustering of recordings within participants*				
	~24-hour evaluation (Grand mean, SD, CI)		In-bed only evaluation (Grand mean, SD, CI)	
	True wake	True sleep	True wake	True sleep
Oakley predicted wake	48.49 (11.68) [46.94, 50.03]	01.39 (01.23) [01.22, 01.55]	05.11 (04.04) [04.58, 05.64]	04.03 (03.14) [03.61, 04.44]
Oakley predicted sleep	21.20 (09.20) [19.98, 22.42]	28.93 (07.49) [27.94, 29.92]	06.32 (07.24) [05.37, 07.28]	84.54 (09.61) [83.27, 85.81]
	**True wake**	**True sleep**	**True wake**	**True sleep**
TCN predicted wake	67.39 (08.48) [66.27, 68.51]	01.37 (02.13) [01.09, 01.65]	06.26 (06.20) [05.44, 07.08]	03.75 (05.55) [03.02, 04.49]
TCN predicted sleep	02.29 (02.96) [01.90, 02.68]	28.94 (07.86) [27.90, 29.98]	05.17 (06.53) [04.31, 06.03]	84.81 (11.76) [83.26, 86.37]
*(b) Confusion matrices for the ~24-hour and in-bed intervals computed as mixed-effects means to account for clustering of recordings within participants*				
	~24-hour evaluation (Mixed-effect mean, SDs, CI)		In-bed only evaluation (Mixed-effect mean, SDs, CI)	
	True wake	True sleep	True wake	True sleep
Oakley predicted wake	47.10 (6.08; 10.03) [44.85, 49.31]	1.25 (0.84; 0.81) [0.98, 1.51]	5.24 (2.04; 3.49) [4.50, 6.00]	3.45 (2.46; 1.45) [2.73, 4.16]
Oakley predicted sleep	22.55 (6.67; 6.54) [20.49, 24.67]	28.93 (0.00; 7.49) [27.95, 29.93]	8.36 (6.52; 4.48) [6.48, 10.29]	83.01 (7.29; 7.04) [80.73, 85.20]
	**True wake**	**True sleep**	**True wake**	**True sleep**
TCN predicted wake	67.39 (0.00; 8.48) [66.26, 68.51]	1.63 (1.83; 1.51) [1.07, 2.19]	6.55 (3.56; 5.16) [5.29, 7.82]	4.00 (4.29; 4.00) [2.63, 5.34]
TCN predicted sleep	2.93 (2.20; 2.17) [2.23, 3.61]	28.94 (0.00; 7.86) [27.89, 29.99]	7.05 (6.13; 3.66) [5.29, 8.84]	82.44 (9.35; 8.54) [79.53, 85.37]

TCN, temporal convolutional network.

Grand mean values represent the percentage of predictions in that cell, averaged across recordings regardless of clustering within participants. The standard deviation and 95% confidence interval of these values are represented in parentheses and brackets, respectively.

Mixed-effect mean values are derived by fitting mixed-effects models separately to each condition with a fixed-effect of intercept, and a random-effect of intercept grouped within participants. Standard deviation values are at the level of the cluster, and residual. Note that in some cases the fitted mixed-effect models produced a singular fit with estimated cluster standard deviations near zero. In the mixed-effects case, 95% confidence intervals are computed via percentile bootstrap with 10,000 replicates.

**Table 8:** Discrepancy (bias and MAE) of interval level statistics for ~24-hour and in-bed intervals

Metric	Measure	Oakley (Grand)	TCN (Grand)	Oakley (Mixed)	TCN (Mixed)	Difference	DF	t	*p*	d	Random effect structure

*~24-hour interval evaluation (bias and MAE)*											
TST (min)	Bias	272.22 (135.97)	12.82 (52.36)	289.38 (104.06; 90.92)	**18.56 (40.79; 36.72)**	− 259.40	389.79	− 33.30	<.01	2.50	Intercept
TST (min)	MAE	272.75 (134.89)	35.22 (40.76)	289.67 (103.49; 90.40)	**39.98 (25.24; 33.28)**	− 237.53	390.40	− 29.65	<.01	2.36	Intercept
*In-bed interval evaluation (bias and MAE)*											
SE (%)	Bias	1.66 (8.76)	1.41 (9.10)	4.10 (8.53; 4.71)	3.56 (8.08; 5.67)	− 0.34	31.84	− 0.53	.599	0.03	Slope and intercept
SOL (min)	Bias	− 13.34 (22.20)	− 0.45 (12.64)	− 12.62 (10.60; 19.25)	**− 0.87 (5.10; 11.76)**	12.88	348.46	7.57	<.01	0.71	Intercept
WASO (min)	Bias	0.25 (43.87)	− 13.91 (40.36)	**− 13.88 (34.92; 27.84)**	− 23.62 (33.90; 26.79)	− 14.16	386.25	− 5.44	<.01	0.32	Intercept
TST (min)	Bias	13.64 (43.49)	8.97 (45.97)	25.35 (37.69; 25.25)	15.62 (39.31; 29.42)	− 6.13	30.64	− 1.73	.093	0.13	Slope and intercept
SE (%)	MAE	6.02 (6.56)	5.97 (7.01)	7.19 (6.38; 4.02)	6.96 (5.91; 4.90)	− 0.06	348.91	− 0.13	.896	0.01	Intercept
SOL (min)	MAE	13.45 (22.13)	6.92 (10.58)	12.72 (10.53; 19.22)	**7.09 (5.10; 9.41)**	− 6.53	349.72	− 4.07	<.01	0.38	Intercept
WASO (min)	MAE	29.96 (31.98)	26.42 (33.50)	31.75 (20.64; 25.58)	33.17 (26.36; 24.34)	− 1.90	45.17	− 0.71	.484	0.05	Slope and intercept
TST (min)	MAE	30.23 (34.06)	30.58 (35.43)	35.60 (29.16; 22.58)	35.46 (26.69; 25.91)	0.35	387.49	0.15	.879	0.01	Intercept

MAE, mean absolute error; SE, sleep efficiency; SOL, sleep onset latency; TCN, temporal convolutional network; TST, total sleep time; WASO, wake after sleep onset.

Bolded values indicate the more favorable outcomes for comparisons that are statistically significant at an alpha level of .05.

Mixed-effects models compared the Oakley algorithm versus TCN model classification, with days nested within participants.

Grand mean values represent the percentage of predictions in that cell, averaged across recordings regardless of clustering within participants. The standard deviation of these values is represented in parentheses.

Mixed-effect mean values are derived by fitting mixed-effects models separately to each condition with a fixed-effect of intercept, and a random-effect of intercept grouped within participants. Standard deviation values are at the level of the cluster, and residual.

Difference is the condition difference estimated from the mixed-effects model used to compute inferential statistics. This value is similar but not necessarily the same as the difference between the mixed-effect condition means, which are estimated independently.

The EcoSleep dataset was excluded from comparisons of SOL or SE, as the method of imputing in-bed labels for the EcoSleep data cannot identify pre-sleep wakefulness within the in-bed interval.

**Table 9:** Discrepancy (CCC and CCCLON) of interval level statistics for ~24-hour and in-bed intervals

Metric	Measure	Oakley	TCN	Difference	Difference excludes 0	Incomplete bootstrap samples

*~24-hour interval evaluation (CCC and CCCLON)*						
TST	CCC	0.18 [0.14, 0.22]	**0.88 [0.85, 0.91]**	− 0.70 [−0.74, −0.66]	*	
TST	CCCLON	0.06 [0.01, 0.11]	**0.71 [0.59, 0.81]**	− 0.65 [−0.75, −0.48]	*	24
*In-bed interval evaluation (CCC and CCCLON)*						
SE	CCC	0.34 [0.23, 0.45]	**0.50 [0.38, 0.59]**	− 0.15 [− 0.29, − 0.04]	*	
SOL	CCC	0.05 [0.02, 0.09]	**0.84 [0.79, 0.88]**	− 0.79 [−0.88, −0.65]	*	
WASO	CCC	0.34 [0.23, 0.45]	0.46 [0.36, 0.55]	− 0.12 [− 0.26, 0.02]		
TST	CCC	0.91 [0.88, 0.93]	0.91 [0.88, 0.93]	0.00 [− 0.02, 0.02]		
SE	CCCLON	0.29 [0.08, 0.48]	0.41 [0.22, 0.57]	− 0.12 [− 0.27, 0.00]		1123
SOL	CCCLON	0.07 [−0.08, 0.21]	**0.82 [0.75, 0.87]**	− 0.75 [−0.89, −0.52]	*	501
WASO	CCCLON	0.26 [0.07, 0.43]	0.29 [0.13, 0.44]	− 0.03 [− 0.24, 0.17]		2307
TST	CCCLON	0.77 [0.66, 0.85]	0.76 [0.65, 0.84]	0.01 [− 0.05, 0.09]		0

CCC, concordance correlation coefficient, calculated across days without respect to clustering within participants; CCCLON, concordance correlation coefficient with longitudinal repeated measures, calculated across days while accounting for clustering of days within participants; SE, sleep efficiency; SOL, sleep onset latency; TCN, temporal convolutional network; TST, total sleep time; WASO, wake after sleep onset.

Asterisks within the column “Difference excludes 0” indicate that the 95% confidence interval of the difference excludes 0. In these cases, bolded values indicate the more favorable outcomes.

The CCCLON internally fits a mixed-effects model to the data to obtain variance components. Some permutations of the bootstrap can produce data sets that can’t be appropriately fit with a mixed-effects model (eg, non-negative approximate variance-covariance). The column Incomplete Bootstrap Samples records the number of instances in which this occurred (out of 10,000).

The EcoSleep dataset was excluded from comparisons of SOL or SE, as the method of imputing in-bed labels for the EcoSleep data cannot identify presleep wakefulness within the in-bed interval.

