# Supplementary material for: Corrigendum to “Performance of an open machine learning model to classify sleep/wake from actigraphy across ~24-hour intervals without knowledge of rest timing” [Sleep Health 9 (2023) 596–610]
Source: Sleep Health. Author manuscript; Available in PMC 2025 Aug 12. (PMC12341005; doi:10.1016/j.sleh.2023.11.008)
Supplement: 1 [file NIHMS2082193-supplement-1.docx]

**Performance of an open machine learning model to classify sleep/wake from actigraphy across ~24-hour intervals without knowledge of rest timing.**

**Supplemental Material**

**1. Supplemental Methods**

**1a. Temporal alignment of Activity Counts and PSG Staging**

Activity counts and sleep/wake labels were initially aligned via their timestamps, by associating the start time of each staged epoch with the nearest actigraphy epoch. If no activity epochs were recorded within half an epoch from a given staged epoch, the activity count for that epoch was considered missing. In the case of two activity counts being equidistant from a given staged epoch (i.e., if the two time series were offset by exactly 15 seconds), the earlier of the two activity counts was associated with the PSG epoch.

Following initial association between PSG staging and actigraphy, the possibility of timing offset between staged epochs and activity counts was evaluated. Actigraphy count timestamps were shifted in one-epoch increments through +- 10 minutes, with a measure of correspondence between PSG staging and actigraphy counts computed at each shift. Within the PSG staging, periods of contiguous wake that were 20 epochs or less in length were first identified, assuming that isolated periods of wakefulness should be associated with movement activity. Using the timestamps of these isolated periods of wake, the proportion of corresponding actigraphy epochs where the actigraphy activity count was greater than or equal to an activity count threshold of 15 was computed. The proportions at each non-zero epoch lag was compared to the proportion at zero lag, and was statistically evaluated using Boschloo’s exact test for 2 x 2 contingency tables^1^ as implemented within SciPy^2^. A non-zero lag was selected if it both produced a greater proportion of above threshold actigraphy epochs in comparison to zero-lag and if the count of above threshold epochs was significant at a two-tailed, .05 significance threshold. In the case of a non-zero lag, the actigraphy timestamps were adjusted to compensate. If no non-zero lags met these criteria, the timestamps of actigraphy data were not adjusted. If a recording did not contain any isolated periods of wakefulness meeting the criteria for selection, the actigraphy count alignment algorithm could not proceed and actigraphy timestamps were not adjusted. The reference from which this procedure was adapted^3^ searched for lags in a +- 5 minute window, identified periods of contiguous wake that were 3 epochs or less in length, and used scan statistics to identify significant lags. Here, the time window to search, and the number of contiguous wake epochs to consider, were extended as visual inspection suggested that some records had offsets as long as 8 minutes that weren’t identified when considered only contiguous epochs that were 3 or less in length. Boschloo’s exact test was used in lieu of a scan statistic due to the former being more commonly implemented in statistical software.

Within this procedure, as epochs missing staging information could potentially be wake, missing epochs bordering wake epochs were considered to contribute towards the count of adjacent wake epochs. For example, a sequence of 20 ‘wake’ epochs followed by a missing epoch would not be considered a valid sequence of 20 or fewer awake epochs, as the participant could have been awake in the subsequent missing epoch. However, following the identification of valid wake sequences, data from missing epochs were excluded from subsequent steps within the alignment procedure. Similarly, actigraphy counts from epochs that were considered ‘missing’ actigraphy data (including epochs where the device was off-wrist) were excluded from the alignment procedure and did not count towards the sum of epochs above or below the activity count threshold.

**1b. Additional Description of Epoch-By-Epoch Performance Metrics**

A variety of metrics are included as each reflects a different (but not necessarily independent) view of model performance. Sleep is considered the positive class, and wake is considered the negative class. With two-classes, classifications can be considered as a member of one of four categories depending on the true and classified class membership. These categories include true positives – a sleep epoch classified as sleep, true negatives – a wake epoch classified as wake, false positives – a wake epoch classified as sleep, and false negatives – a sleep epoch classified as wake. The proportion of epochs within each of these categories are then used to derive the classification-based metrics. Except for PABAK, all metrics were computed using functions from the Scikit-learn^4^ (ver. 1.1.2) package for Python, according to standard definitions.^5^ As this package does not include an implementation of PABAK, this was calculated as (2 * accuracy) – 1 to map accuracy from the range 0,1 to -1,1 ^6^.

**1c. Sleep Metric Definitions**

Across the ~24-Hour interval, total sleep time (TST) was calculated. Across the in-bed interval, TST, sleep onset latency (SOL), sleep efficiency (SE), and wake after sleep onset were calculated. These metrics follow standard definitions:

TST: The total number of minutes classified as sleep across the interval.

SOL: The latency in minutes between the beginning of the in-bed interval and the first epoch classified as sleep.

SE: Within the in-bed interval, the ratio of the sum of epochs classified as sleep to the sum of epochs classified as sleep or wake, expressed as a percentage. Note that epochs that weren’t classified due to missing data are excluded from the ratio.

WASO: Within the in-bed interval, the number of minutes classified as wake between sleep onset (the first sleep epoch) and sleep offset (the last sleep epoch). Note that this does not include terminal wakefulness (time awake in bed after the final awakening), which has been included in WASO by some researchers.

**1d. Additional Description of Interval Level Discrepancy Measures**

Several discrepancy measures were included as they differ in their definition of classifier error. Bias is calculated for each measure by calculating the predicted (classifier) minus true (PSG) values for each record within the interval and averaging the result. This value is in the original units of the metric and is typically included within Bland-Altman plots (unless proportional bias is plotted instead). However, the bias of the classifier on an outcome measure does not necessarily reflect the average error of individual records, as for example symmetric errors across different records would average to a bias of 0. The MAE is calculated by again calculating the predicted (classifier) minus true (PSG) values from each day (or night) but taking the absolute value prior to averaging the result. This reflects the average unsigned error in the original units of the metric, but does not indicate the degree of bias, if any. The CCC is measure of agreement, capturing the linear relationship between the true and predicted values, but unlike Pearson correlation, also incorporates the degree of bias. CCC can be conceptualized as reflecting the deviation of predicted values from the 45° line of identity on a scatterplot of true vs predicted values.^7^ Like Pearson correlation, the CCC ranges from -1 to 1, and does not represent error in the original units of the metric.

**1e. Evaluation of TCN Classifier on the MESA Dataset**

MESA data was used for model testing only, no MESA data was used for model training or tuning. The MESA dataset was split into five partitions, with each partition tested using the trained model from one of the five model development folds (see Data Partitions within the main manuscript).

As the MESA dataset contains only one night of data per participant, the mixed-effects models using to evaluate performance differences between TCN and Oakley models only included a random-effect of model intercept, grouped within participants.

The effect-size computation described in the main manuscript was also used for the MESA dataset models. As each MESA participant only contributed one night of data, the resulting value is equal to traditional Cohen’s d (condition difference divided by pooled standard deviation). Additional statistics reported in the main manuscript for reporting the nested ~24-Hour data, such as CCCLON or mixed-effects derived means, were not included (as the MESA data was not nested).

Bland-Altman plots are additionally plotted for the MESA dataset. Unlike the results presented within the main manuscript, the MESA dataset is not nested – each participant contributes only 1 night of data. Accordingly, a different approach was used for calculating the limits of agreement. Limits of agreement computation was adapted from the “BAplot” function within the “Analytical pipeline and functions for testing the performance of sleep-tracking technology” code (<https://github.com/SRI-human-sleep/sleep-trackers-performance>; git commit e985ed3, 03/21/2022) from Menghini and colleagues.^8^ Internal to this function, proportional bias is evaluated by fitting a linear regression between ground truth and the classifier – ground truth differences. If this regression coefficient is significant (according to a bootstrapped 95% confidence interval with 10,000 replicates), proportional bias and limits of agreement are plotted, otherwise fixed bias and limits of agreement are plotted. In addition, 95% confidence intervals around bias and limits of agreement are calculated using a basic bootstrap with 10,000 replicates.

**1f. Alternate Model with Causal Convolutions**

The analysis presented within the main manuscript uses a convolution kernel which operates bidirectionally – the sleep/wake classification for a given epoch depends on the actigraphy counts in both the past and the future. This structure is usable if analysis is to be performed following data collection, however the use of future epochs precludes classifications from being provided in real time as data is collected.

We additionally trained a model using only ‘causal’ convolutions, where the classification of a given epoch depends only on the actigraphy counts at the current or prior epochs. The range of hyperparameters for this causal model is included in Table S1, with the optimal hyperparameters identified included in Table S2. As the convolutions only operate on the current and prior epochs, the range of kernel sizes has been decreased to operate on the same number of current and prior epochs as the bidirectional model. For example, the maximum kernel size of the bidirectional model was 7, which includes the current epoch, 3 epochs prior, and 3 epochs future. The maximum kernel size of the causal model is instead 4, including the current epoch and 3 epochs prior.

Table S1. Hyperparameters to tune, with the range of values to search for each, for the causal model.

| **Hyperparameter** | **Potential Values** |
| --- | --- |
| Number of Filters in TCN | 1, 2, 4, 8, 16, 32, 64 |
| TCN Kernel Size | 1, 2, 3, 4 |
| Set of TCN Dilations | [ 1, 2], [ 1, 2, 4], [ 1, 2, 4, 8], [ 1, 2, 4, 8, 16,], [ 1, 2, 4, 8, 16, 32] |
| Dropout Rate Inside TCN | 0.0, 0.1, 0.2, 0.3, 0.4, 0.5 |
| Normalization | layer normalization, batch normalization, none |
| Dropout Rate Prior to Dense Layer | 0.0, 0.1, 0.2, 0.3, 0.4, 0.5 |
| Learning Rate | 0.0001 to 0.01 with log sampling |

**Notes:**

TCN = Temporal convolutional network

Table S2. Optimal hyperparameters identified within each fold of the model, for the causal model.

| **Hyperparameter** | **Fold 1** | **Fold 2** | **Fold 3** | **Fold 4** | **Fold 5** |
| --- | --- | --- | --- | --- | --- |
| Number of Filters in TCN | 64 | 64 | 64 | 64 | 64 |
| TCN Kernel Size | 4 | 4 | 4 | 4 | 4 |
| Set of TCN Dilations | [ 1, 2, 4, 8, 16, 32] | [ 1, 2, 4, 8, 16, 32] | [ 1, 2, 4, 8, 16, 32] | [ 1, 2, 4, 8, 16, 32] | [ 1, 2, 4, 8, 16, 32] |
| Dropout Inside TCN | 0 | 0 | 0 | 0.2 | 0 |
| Normalization | Layer Normalization | Layer Normalization | None | None | None |
| Dropout Prior to Dense Layer | 0.5 | 0.3 | 0.5 | 0.2 | 0.5 |
| Learning Rate | 6.49E-04 | 8.66E-04 | 2.79E-04 | 7.72E-04 | 6.35E-04 |

**Notes:**

TCN = Temporal convolutional network

**1g. Performance Relative to Alternative Classifiers**

The analyses presented within the main manuscript used the Oakley classifier as a point of reference for TCN classifier performance. The Oakley classifier was used because we believe it is the algorithm most commonly used to classify data from Actiwatch Spectrum devices. However, as alternative algorithms are also in use, we have additionally compared the bidirectional TCN classifier presented with the main manuscript to the Sadeh^9^ and Scripps Clinic^10^ algorithms. These classifications were generated using the implementations of the algorithms within the PyActigraphy^11^ package (ver. 1.2) for Python. While the Cole-Kripke^12^ algorithm is also commonly used, it was not evaluated here. As the Cole-Kripke algorithm produces sleep/wake classifications with a resolution of a 60-second epoch, it complicates comparison to our ground truth or alternative classifier sleep/wake values, which all use a 30-second epoch.

**2. Supplemental Results:**

**2a. Evaluation of TCN Classifier on the MESA Dataset**

**MESA Epoch-by-epoch Performance**

Epoch-by-epoch classification performance for the MESA dataset is indicated within Table S3. When evaluating all available data, the TCN model produces favorable epoch-by-epoch performance to the Oakley classifications on accuracy, balanced accuracy, specificity, PPV, MCC, and PABAK, while the Oakley classifier is favored on sensitivity, NPV and F1-score. When restricting the performance evaluation to only the known in-bed’ interval, the TCN shows favorable epoch-by-epoch performance on balanced accuracy, specificity, and PPV, while the Oakley classifier is preferred on accuracy, sensitivity, NPV, F1-score, and PABAK.

Figure S1 displays the ROC curve for the TCN classifier, separately for all-available and in-bed intervals. The ROC curve depicts the trade-off between the true positive rate (sensitivity) and the false positive rate (1 – specificity) as the probability threshold for classification is altered. The model performs more favorably across the all-available interval than the in-bed interval, also reflected numerically by the AUC values in Table S3. Table S4 displays a confusion matrix for the performance of both the Oakley and TCN classifiers on the MESA dataset, at both evaluation intervals. The confusion matrices reiterate the increased specificity for the TCN model that had been demonstrated statistically in Table S3. In addition, by summing within columns, the base rates of sleep and wake within each interval can be obtained.

Table S3. Epoch-level classification performance for all-available and in-bed intervals both the Oakley and TCN classifiers on the MESA dataset.

MESA All-Available Interval Evaluation (Epoch-by-Epoch)

| Metric | Oakley (Grand) | TCN (Grand) | Difference | DF | t | p | d | Random Effect Structure |
| --- | --- | --- | --- | --- | --- | --- | --- | --- |
| AUC |  | 0.866 (0.122) |  |  |  |  |  |  |
| Accuracy | 0.791 (0.102) | **0.803 (0.139)** | 0.012 | 1,689 | 3.91 | <.01 | 0.10 | intercept |
| Balanced Accuracy | 0.694 (0.091) | **0.758 (0.125)** | 0.064 | 1,689 | 25.91 | <.01 | 0.58 | intercept |
| Sensitivity | **0.960 (0.047)** | 0.843 (0.231) | -0.117 | 1,689 | -22.64 | <.01 | 0.70 | intercept |
| Specificity | 0.428 (0.177) | **0.672 (0.241)** | 0.245 | 1,689 | 52.42 | <.01 | 1.15 | intercept |
| PPV | 0.780 (0.126) | **0.849 (0.153)** | 0.069 | 1,689 | 23.52 | <.01 | 0.49 | intercept |
| NPV | **0.821 (0.181)** | 0.753 (0.234) | -0.068 | 1,689 | -15.15 | <.01 | 0.32 | intercept |
| F1-Score | **0.854 (0.091)** | 0.823 (0.193) | -0.031 | 1,689 | -7.74 | <.01 | 0.21 | intercept |
| MCC | 0.476 (0.180) | **0.551 (0.245)** | 0.075 | 1,689 | 16.77 | <.01 | 0.35 | intercept |
| PABAK | 0.582 (0.204) | **0.606 (0.279)** | 0.024 | 1,689 | 3.91 | <.01 | 0.10 | intercept |

MESA In-Bed Interval Evaluation (Epoch-by-Epoch)

| Metric | Oakley (Grand) | TCN (Grand) | Difference | DF | t | p | d | Random Effect Structure |
| --- | --- | --- | --- | --- | --- | --- | --- | --- |
| AUC |  | 0.822 (0.137) |  |  |  |  |  |  |
| Accuracy | **0.820 (0.104)** | 0.790 (0.151) | -0.030 | 1,689 | -9.26 | <.01 | 0.23 | intercept |
| Balanced Accuracy | 0.673 (0.094) | **0.695 (0.124)** | 0.022 | 1,689 | 9.20 | <.01 | 0.20 | intercept |
| Sensitivity | **0.960 (0.047)** | 0.843 (0.231) | -0.117 | 1,689 | -22.63 | <.01 | 0.70 | intercept |
| Specificity | 0.387 (0.184) | **0.547 (0.280)** | 0.161 | 1,689 | 29.67 | <.01 | 0.68 | intercept |
| PPV | 0.826 (0.123) | **0.857 (0.151)** | 0.032 | 1,689 | 11.27 | <.01 | 0.23 | intercept |
| NPV | **0.741 (0.213)** | 0.653 (0.267) | -0.089 | 1,689 | -16.97 | <.01 | 0.37 | intercept |
| F1-Score | **0.882 (0.086)** | 0.828 (0.193) | -0.055 | 1,689 | -13.66 | <.01 | 0.37 | intercept |
| MCC | 0.434 (0.187) | 0.433 (0.242) | -0.001 | 1,689 | -0.26 | 0.796 | 0.01 | intercept |
| PABAK | **0.640 (0.209)** | 0.580 (0.302) | -0.059 | 1,689 | -9.26 | <.01 | 0.23 | intercept |

**Notes:**

MESA = Multi-Ethnic Study of Atherosclerosis

TCN = Temporal convolutional network

AUC = Area under the receiver operating characteristic curve

PABAK = Prevalence-adjusted and bias-adjusted kappa

PPV = Positive predictive value

NPV = Negative predictive value

MCC = Matthews correlation coefficient

TCN = Temporal Convolution Network

DF = Mixed-effects model degrees of freedom

t = Mixed-effects model t-value for the comparison between classifiers

Bolded values indicate the more favorable outcomes for comparisons that are statistically significant at an alpha level of .05.

AUC not possible for Oakley classifier given discrete classification.

Grand mean values are averaged across recordings (MESA data was not nested). The standard deviation of these values is represented in parentheses.


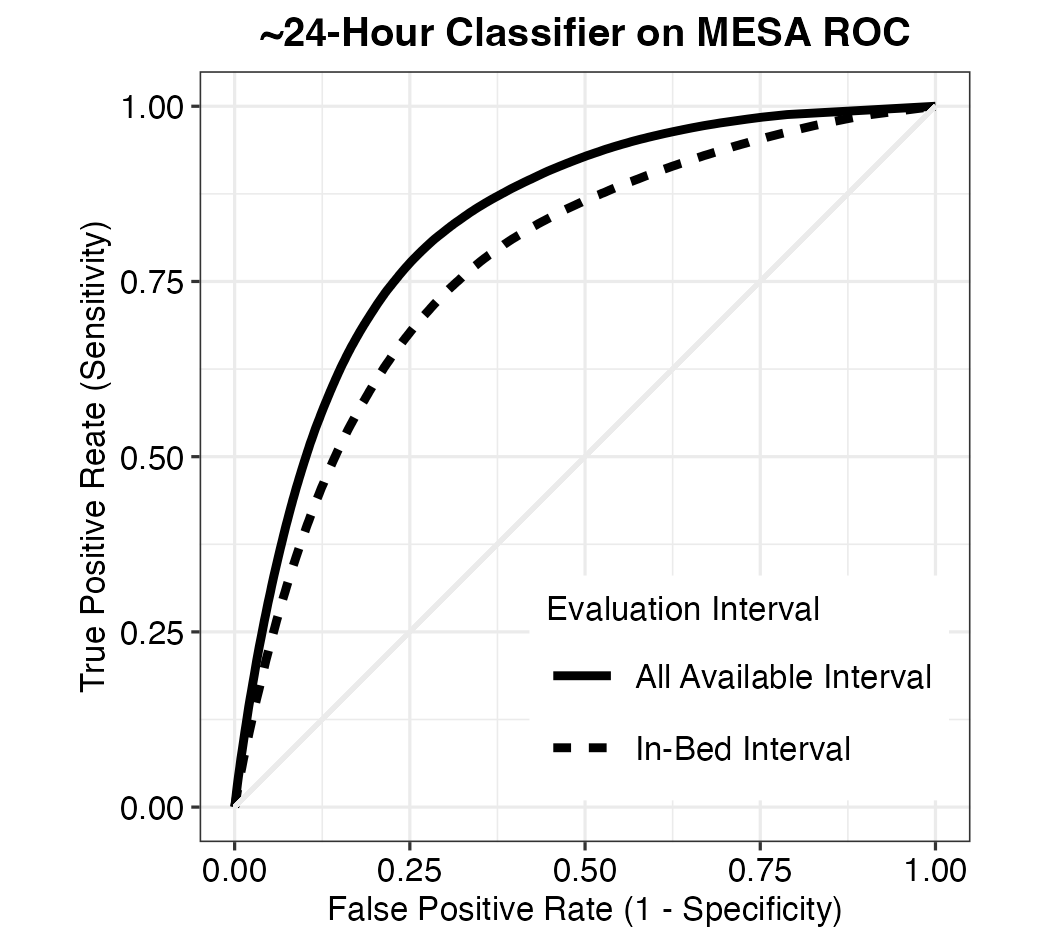


Figure S1: Receiver operating characteristic (ROC) for classifier performance on the Multi-Ethnic Study of Atherosclerosis (MESA) data set. Performance is separately displayed for evaluation across the full interval (solid line) or only within the in-bed interval (dashed line). Each line is the mean of ROC curves from individual days or nights, combined via threshold averaging.^13^

Table S4. Confusion matrices for the Multi-Ethnic Study of Atherosclerosis (MESA) data set, for both the All-Available and In-Bed intervals, for both the Oakley and temporal convolutional network (TCN) classifiers.

|  | **MESA All-Available Evaluation** |  |  | **MESA In-Bed Evaluation** |  |
| --- | --- | --- | --- | --- | --- |
|  |  |  |  |  |  |
|  | True Wake | True Sleep |  | True Wake | True Sleep |
| Oakley Predicted Wake | 13.39 (07.91) [13.01, 13.76] | 02.68 (03.08) [02.54, 02.83] |  | 08.93 (06.50) [08.62, 09.24] | 02.97 (03.40) [02.81, 03.13] |
| Oakley Predicted Sleep | 18.21 (10.05) [17.73, 18.68] | 65.73 (13.37) [65.09, 66.36] |  | 15.04 (10.16) [14.56, 15.53] | 73.06 (13.54) [72.41, 73.70] |
|  |  |  |  |  |  |
|  | True Wake | True Sleep |  | True Wake | True Sleep |
| TCN Predicted Wake | 21.94 (13.26) [21.31, 22.57] | 10.03 (14.41) [09.34, 10.72] |  | 14.06 (12.48) [13.46, 14.65] | 11.07 (15.79) [10.31, 11.82] |
| TCN Predicted Sleep | 09.65 (08.58) [09.24, 10.06] | 58.38 (20.14) [57.42, 59.34] |  | 09.91 (08.79) [09.49, 10.33] | 64.96 (21.73) [63.93, 66.00] |

**Notes:**

Values represent the percentage of predictions in that cell, averaged across recordings. The standard deviation and 95% confidence interval of these values are represented in parentheses and brackets, respectively.

**MESA Sleep Metric Discrepancy Performance at All-Available and In-Bed Intervals**

Discrepancy of classifiers within the All-Available and In-Bed intervals for the MESA dataset are indicated in Table S5 for bias and MAE, and Table S6 for CCC. As the MESA dataset only contains 1 day per participant, only CCC is reported, not both CCC and CCCLON as reported for the nested ~24-hour data in the main manuscript. The TCN classifier failed to capture any sleep time for 23 of the 1,690 records evaluated. With no sleep onset, SOL and WASO are not defined for those records for the TCN classifier. To compare on the same data, those 23 records were also not analyzed for SOL or WASO for the Oakley classifier.

Across all-available data, relative to the Oakley classifications, the amount of TST predicted by the TCN model more closely matches the ground truth in terms of bias, MAE, or CCC. Scatterplots and Bland-Altman plots visualizing the relationship between true and predicted values for TST across the all-available interval are shown in Figure S2.

Looking exclusively within the time limits of the In-Bed interval, the TCN has favorable performance in bias on SE and bias on TST, however the Oakley classifier is favorable in terms of bias on SOL and favorable in MAE on all metrics. The TCN is favorable in terms of CCC of SE or SOL, while the two classifiers did not significantly differ in CCC of WASO or TST. Scatterplots and Bland-Altman plots visualizing the relationship between true and predicted values for these metrics across the in-bed interval are shown in Figure S3.

Table S5. Discrepancy (bias and MAE) of interval level statistics for All-Available and in-bed intervals for the Multi-Ethnic Study of Atherosclerosis (MESA) dataset.

MESA All-Available Interval Evaluation (Bias and MAE)

| Metric | Measure | Oakley (Grand) | TCN (Grand) | Difference | DF | t | p | d | Random Effect Structure |
| --- | --- | --- | --- | --- | --- | --- | --- | --- | --- |
| TST (Minutes) | Bias | 83.85 (61.91) | **-0.26 (101.12)** | -84.11 | 1,689 | -35.94 | <.01 | 1.00 | intercept |
| TST (Minutes) | MAE | 85.22 (60.01) | **70.19 (72.78)** | -15.03 | 1,689 | -7.77 | <.01 | 0.23 | intercept |

MESA In-Bed Interval Evaluation (Bias and MAE)

| Metric | Measure | Oakley (Grand) | TCN (Grand) | Difference | DF | t | p | d | Random Effect Structure |
| --- | --- | --- | --- | --- | --- | --- | --- | --- | --- |
| SE (%) | Bias | 12.07 (10.98) | **-1.15 (20.64)** | -13.23 | 1,689 | -28.14 | <.01 | 0.80 | intercept |
| SOL (Minutes) | Bias | **-19.36 (35.68)** | 30.77 (71.34) | 50.13 | 1,666 | 28.46 | <.01 | 0.89 | intercept |
| WASO (Minutes) | Bias | -39.77 (54.97) | -40.51 (69.87) | -0.74 | 1,666 | -0.53 | 0.599 | 0.01 | intercept |
| TST (Minutes) | Bias | 58.91 (55.86) | **-4.11 (98.51)** | -63.01 | 1,689 | -27.79 | <.01 | 0.79 | intercept |
| SE (%) | MAE | **12.69 (10.26)** | 14.16 (15.05) | 1.47 | 1,689 | 3.82 | <.01 | 0.11 | intercept |
| SOL (Minutes) | MAE | **20.07 (35.28)** | 40.03 (66.59) | 19.96 | 1,666 | 11.01 | <.01 | 0.37 | intercept |
| WASO (Minutes) | MAE | **48.65 (47.28)** | 59.67 (54.42) | 11.01 | 1,666 | 10.25 | <.01 | 0.22 | intercept |
| TST (Minutes) | MAE | **61.62 (52.85)** | 67.36 (71.97) | 5.74 | 1,689 | 3.18 | <.01 | 0.09 | intercept |

**Notes:**

TCN = Temporal convolutional network

MAE = mean absolute error

SE = Sleep efficiency

SOL = Sleep onset latency

WASO = Wake after sleep onset

TST = Total sleep time

Grand mean values are averaged across recordings (MESA data was not clustered). The standard deviation of these values is represented in parentheses.

Bolded values indicate the more favorable outcomes for comparisons that are statistically significant at an alpha level of .05.

Table S6. Discrepancy (CCC) of interval level statistics for All-Available and in-bed intervals for the Multi-Ethnic Study of Atherosclerosis (MESA) dataset.

MESA All-Available Interval Evaluation (CCC)

| Metric | Measure | Oakley | TCN | Difference | Difference Excludes 0 |
| --- | --- | --- | --- | --- | --- |
| TST | CCC | 0.45 [0.43, 0.48] | **0.55 [0.52, 0.58]** | -0.09 [-0.13, -0.06] | * |

MESA In-Bed Interval Evaluation (CCC)

| Metric | Measure | Oakley | TCN | Difference | Difference Excludes 0 |
| --- | --- | --- | --- | --- | --- |
| SE | CCC | 0.29 [0.27, 0.32] | **0.41 [0.38, 0.45]** | -0.12 [-0.16, -0.08] | * |
| SOL | CCC | 0.07 [0.06, 0.09] | **0.20 [0.17, 0.23]** | -0.13 [-0.18, -0.07] | * |
| WASO | CCC | 0.32 [0.29, 0.35] | 0.30 [0.26, 0.33] | 0.02 [-0.03, 0.09] |  |
| TST | CCC | 0.59 [0.57, 0.62] | 0.56 [0.53, 0.59] | 0.03 [-0.01, 0.06] |  |

**Notes:**

TCN = Temporal convolutional network

CCC = concordance correlation coefficient

SE = Sleep efficiency

SOL = Sleep onset latency

WASO = Wake after sleep onset

TST = Total sleep time

Bolded values indicate the more favorable outcomes for comparisons with a 95% confidence interval of the difference excluding 0.


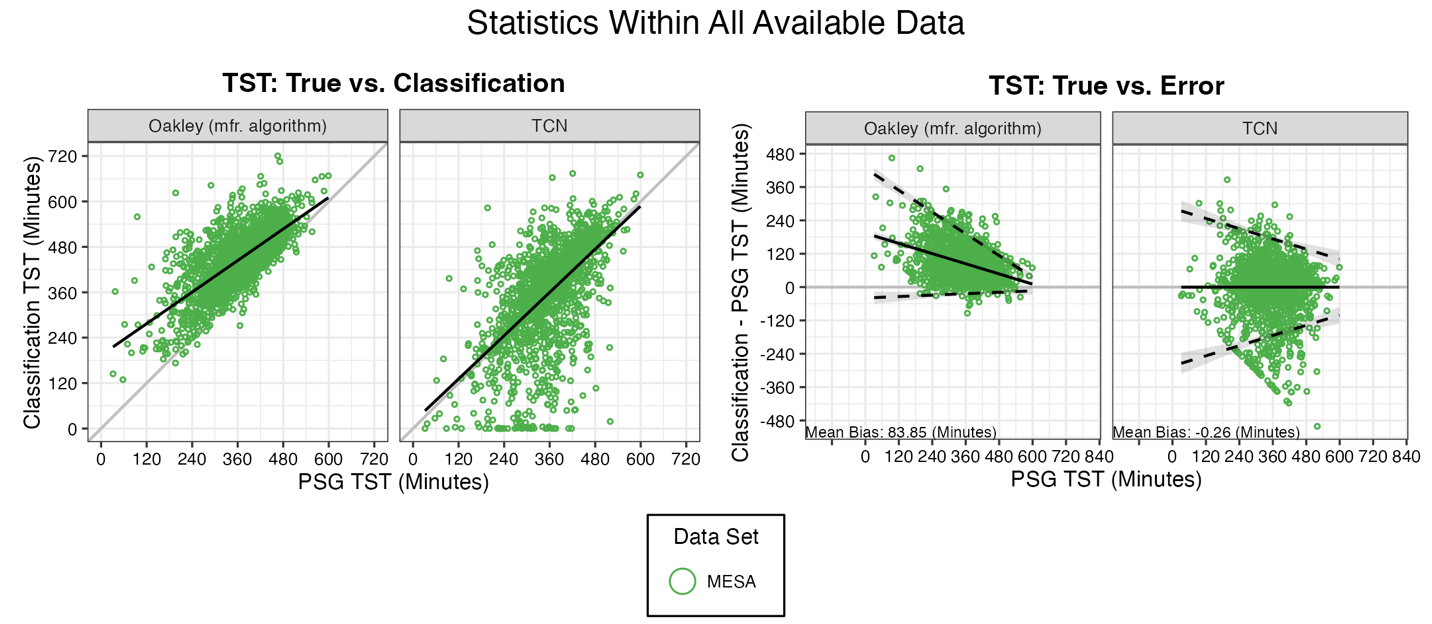


Figure S2: PSG-derived total sleep time (TST) over All-Available data for the Multi-Ethnic Study of Atherosclerosis (MESA) dataset, compared to the Oakley classifier or our temporal convolutional network (TCN) classifier. Left column shows PSG vs classification for each classification type, with linear regression of predicted TST on true TST for reference (black line). Right column shows Bland-Altman PSG vs. classification error (classification TST – PSG TST). Proportional bias was determined to be present for the Oakley classifier, but not the TCN classifier. Indicated are bias (black line) and the upper and lower limits of the 95% limits of agreement (dashed black lines) with 95% confidence intervals for each shaded in gray.


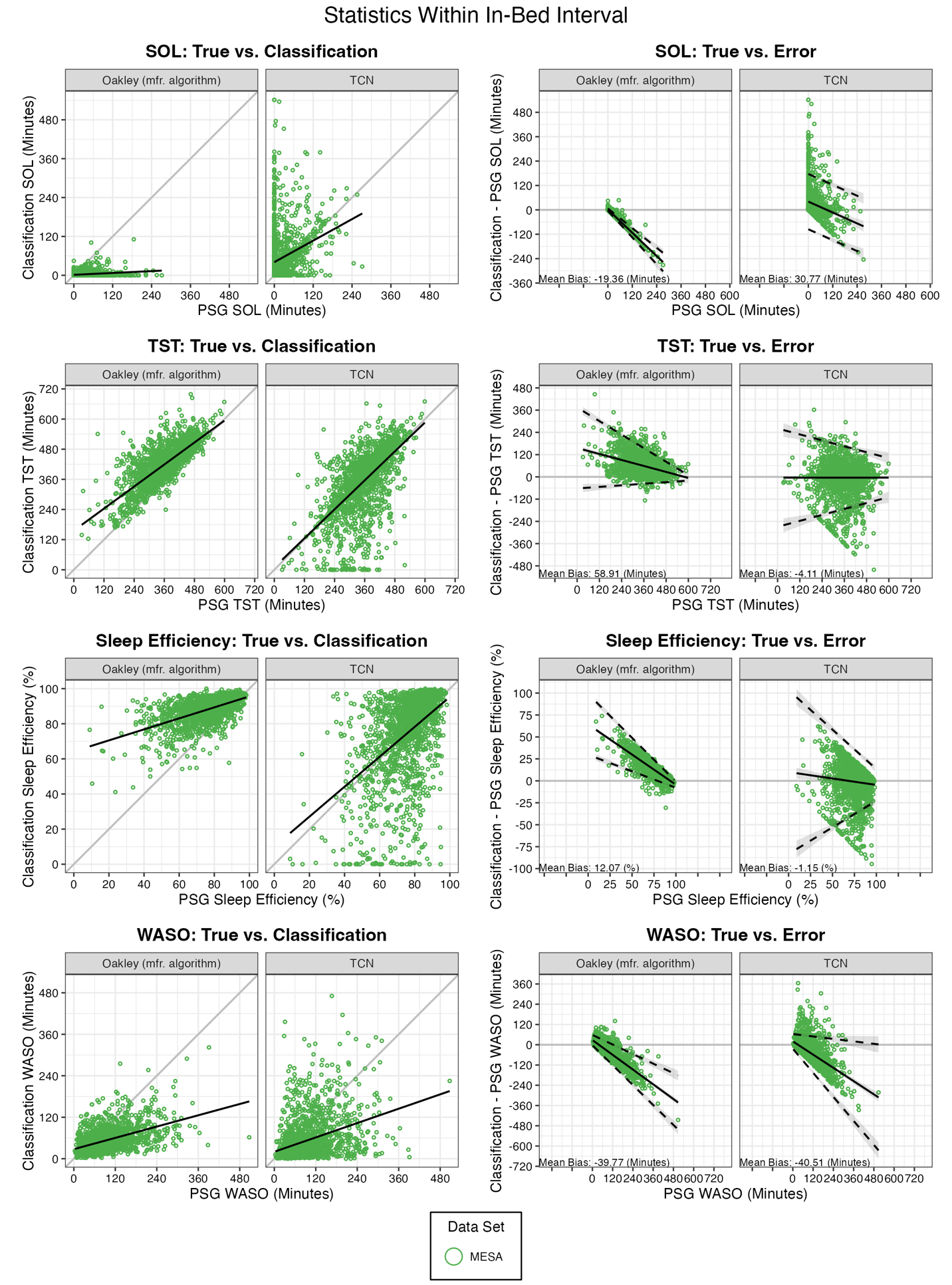


Figure S3: PSG-derived sleep metrics for the Multi-Ethnic Study of Atherosclerosis (MESA) dataset, restricted to the In-Bed interval (as defined by RPSGT), compared to values from the Oakley classification outputs or our temporal convolutional network (TCN) classifier restricted to the same interval. Left column shows PSG vs classification for each classification type, with linear regression of predicted metric on true metric for reference (black line). Right column shows a Bland-Altman plot of PSG metric vs. classification metric error (classification metric – PSG metric). Plotted are sleep onset latency (SOL), total sleep time (TST), sleep efficiency (SE), and wake after sleep onset (WASO). Proportional bias was not present for TST for the TCN classifier but was determined to be present for TST for the Oakley classifier, and for all other metrics, for both classifiers. Indicated are bias (black line) and the upper and lower limits of the 95% limits of agreement (dashed black lines) with 95% confidence intervals for each shaded in gray.

**2b. Alternate Model with Causal Convolutions**

**~24-Hour Data Performance using Causal TCN**

Epoch-level performance of the alternative model that uses only causal convolutions are presented within Table S7 for the ~24-Hour data, at both ~24-Hour and in-bed intervals. At the ~24 Hour interval, the causal TCN produces favorable epoch-by-epoch performance relative to the Oakley classifier on accuracy, balanced accuracy, specificity, PPV, F1-Score, MCC, and PABAK. The Oakley classifier is favorable on sensitivity, while NPV did not significantly differ. At the in-bed interval, the causal TCN has favorable performance on balanced accuracy, specificity, and PPV, while the Oakley classifier had favorable performance on sensitivity. Accuracy, NPV, F1-Score, MCC, and PABAK did not significantly differ.

Discrepancy of the causal TCN within the ~24-Hour and in-bed intervals are presented within Table S8 for bias and MAE, and Table S9 for CCC/CCCLON. At the ~24-Hour interval, the causal TCN has favorable estimates of TST in terms of bias, MAE, and both CCC and CCCLON. At the in-bed interval, the causal TCN has favorable performance in terms of bias, CCC, and CCCLON of SE, bias, CCC, and CCCLON of SOL, CCC of WASO, and bias of TST. The Oakley classifier had favorable performance in terms of bias of WASO. Note that while the mixed-effects derived condition means suggest that the TCN model may have preferrable WASO bias (closer to 0), the estimated marginal means from the statistical model directly comparing the two classifiers estimates WASO bias from the Spectrum model to be closer to 0 by 5.34 minutes: -11.85 vs. -17.19.

Table S7. Epoch-level classification performance on the ~24-Hour data, for ~24-Hour and In-Bed periods, for the causal model that only uses the current and prior epochs.

~24-Hour Data, ~24-Hour Interval Epoch-by-Epoch Evaluation, Causal Model

| Metric | Oakley (Grand) | TCN (Grand) | Oakley (Mixed) | TCN (Mixed) | Difference | DF | t | p | d | Random Effect Structure |
| --- | --- | --- | --- | --- | --- | --- | --- | --- | --- | --- |
| AUC |  | 0.987 (0.015) |  | 0.983 (0.012; 0.011) |  |  |  |  |  |  |
| Accuracy | 0.774 (0.089) | 0.953 (0.034) | 0.762 (0.062; 0.065) | **0.946 (0.021; 0.028)** | 0.179 | 390.50 | 32.47 | <.01 | 2.64 | intercept |
| Balanced Accuracy | 0.824 (0.063) | 0.946 (0.037) | 0.817 (0.040; 0.050) | **0.940 (0.025; 0.029)** | 0.127 | 43.39 | 17.54 | <.01 | 2.60 | slope and intercept |
| Sensitivity | 0.954 (0.036) | 0.932 (0.068) | **0.959 (0.029; 0.018)** | 0.926 (0.052; 0.050) | -0.031 | 37.62 | -4.45 | <.01 | 0.48 | slope and intercept |
| Specificity | 0.693 (0.134) | 0.960 (0.041) | 0.674 (0.093; 0.099) | **0.954 (0.025; 0.034)** | 0.267 | 392.01 | 33.05 | <.01 | 2.68 | intercept |
| PPV | 0.587 (0.125) | 0.918 (0.083) | 0.578 (0.073; 0.102) | **0.902 (0.060; 0.063)** | 0.334 | 42.80 | 30.30 | <.01 | 3.23 | slope and intercept |
| NPV | 0.972 (0.025) | 0.971 (0.029) | 0.974 (0.014; 0.019) | 0.968 (0.021; 0.022) | -0.003 | 33.45 | -1.08 | 0.287 | 0.10 | slope and intercept |
| F1-Score | 0.719 (0.094) | 0.922 (0.058) | 0.713 (0.055; 0.078) | **0.911 (0.041; 0.046)** | 0.206 | 42.60 | 23.21 | <.01 | 2.73 | slope and intercept |
| MCC | 0.600 (0.116) | 0.891 (0.076) | 0.589 (0.075; 0.092) | **0.875 (0.051; 0.061)** | 0.294 | 41.86 | 23.82 | <.01 | 3.12 | slope and intercept |
| PABAK | 0.548 (0.177) | 0.905 (0.068) | 0.525 (0.125; 0.131) | **0.893 (0.042; 0.056)** | 0.357 | 390.50 | 32.47 | <.01 | 2.64 | intercept |

~24-Hour Data, In-Bed Interval Epoch-by-Epoch Evaluation, Causal Model

| Metric | Oakley (Grand) | TCN (Grand) | Oakley (Mixed) | TCN (Mixed) | Difference | DF | t | p | d | Random Effect Structure |
| --- | --- | --- | --- | --- | --- | --- | --- | --- | --- | --- |
| AUC |  | 0.902 (0.084) |  | 0.874 (0.071; 0.052) |  |  |  |  |  |  |
| Accuracy | 0.897 (0.070) | 0.894 (0.074) | 0.882 (0.060; 0.047) | 0.876 (0.061; 0.051) | -0.002 | 385.18 | -0.44 | 0.657 | 0.03 | intercept |
| Balanced Accuracy | 0.744 (0.109) | 0.781 (0.111) | 0.718 (0.078; 0.076) | **0.751 (0.080; 0.073)** | 0.033 | 50.29 | 3.04 | <.01 | 0.31 | slope and intercept |
| Sensitivity | 0.954 (0.036) | 0.933 (0.067) | **0.960 (0.028; 0.018)** | 0.928 (0.051; 0.050) | -0.029 | 37.00 | -4.28 | <.01 | 0.46 | slope and intercept |
| Specificity | 0.534 (0.232) | 0.629 (0.236) | 0.475 (0.173; 0.150) | **0.573 (0.177; 0.152)** | 0.095 | 47.58 | 4.20 | <.01 | 0.41 | slope and intercept |
| PPV | 0.930 (0.081) | 0.944 (0.070) | 0.907 (0.072; 0.052) | **0.925 (0.064; 0.042)** | 0.015 | 386.07 | 3.40 | <.01 | 0.18 | intercept |
| NPV | 0.549 (0.213) | 0.547 (0.224) | 0.597 (0.147; 0.152) | 0.560 (0.145; 0.173) | -0.022 | 47.53 | -0.99 | 0.329 | 0.10 | slope and intercept |
| F1-Score | 0.939 (0.047) | 0.936 (0.052) | 0.930 (0.041; 0.032) | 0.923 (0.045; 0.036) | -0.003 | 384.53 | -1.06 | 0.291 | 0.06 | intercept |
| MCC | 0.460 (0.142) | 0.505 (0.168) | 0.450 (0.085; 0.115) | 0.476 (0.118; 0.116) | 0.028 | 47.52 | 1.43 | 0.160 | 0.18 | slope and intercept |
| PABAK | 0.793 (0.140) | 0.789 (0.148) | 0.765 (0.121; 0.095) | 0.753 (0.122; 0.103) | -0.004 | 385.18 | -0.44 | 0.657 | 0.03 | intercept |

**Notes:**

TCN = Temporal convolutional network

AUC = Area under the receiver operating characteristic curve

PABAK = Prevalence-adjusted and bias-adjusted kappa

PPV = Positive predictive value

NPV = Negative predictive value

MCC = Matthews correlation coefficient

TCN = Temporal Convolution Network

DF = Mixed-effects model degrees of freedom

t = Mixed-effects model t-value for the comparison between classifiers

Bolded values indicate the more favorable outcomes for comparisons that are statistically significant at an alpha level of .05.

AUC not possible for Oakley classifier given discrete classification.

Mixed-effects models compared the manufacturer’s standard algorithm versus TCN model classification, with days nested within participants.

Grand mean values are averaged across recordings regardless of clustering within participants. The standard deviation of these values is represented in parentheses.

Mixed-effect mean values are derived by fitting mixed-effects models separately to each condition with a fixed-effect of intercept, and a random-effect of intercept grouped within participants. Standard deviation values are at the level of the cluster, and residual.

Difference is the condition difference estimated from the mixed-effects model used to compute inferential statistics. This value is similar but not necessarily the same as the difference between the mixed-effect condition means, which are estimated independently.

For EcoSleep Study, in-bed interval was not set for participants, so nighttime sleep interval imputed from PSG scored start/end of nighttime sleep epochs.

Table S8. Discrepancy (bias and MAE) of interval level statistics for ~24-hour and in-bed intervals, for the causal model that incorporates only the current and prior epochs.

~24-Hour Data, ~24-Hour Interval Evaluation (Bias and MAE), Causal Model

| Metric | Measure | Oakley (Grand) | TCN (Grand) | Oakley (Mixed) | TCN (Mixed) | Difference | DF | t | p | d | Random Effect Structure |
| --- | --- | --- | --- | --- | --- | --- | --- | --- | --- | --- | --- |
| TST (Minutes) | Bias | 272.22 (135.97) | 10.00 (51.73) | 289.38 (104.06; 90.92) | **14.45 (38.12; 37.84)** | -262.21 | 389.59 | -33.54 | <.01 | 2.53 | intercept |
| TST (Minutes) | MAE | 272.75 (134.89) | 35.80 (38.58) | 289.67 (103.49; 90.40) | **38.08 (21.65; 33.31)** | -236.95 | 390.21 | -29.48 | <.01 | 2.36 | intercept |

~24-Hour Data, In-Bed Interval Evaluation (Bias and MAE), Causal Model

| Metric | Measure | Oakley (Grand) | TCN (Grand) | Oakley (Mixed) | TCN (Mixed) | Difference | DF | t | p | d | Random Effect Structure |
| --- | --- | --- | --- | --- | --- | --- | --- | --- | --- | --- | --- |
| SE (%) | Bias | 1.66 (8.76) | -0.94 (8.96) | 4.10 (8.53; 4.71) | **0.84 (7.70; 5.87)** | -2.87 | 28.26 | -4.33 | <.01 | 0.30 | slope and intercept |
| SOL (Minutes) | Bias | -13.34 (22.20) | 6.40 (18.86) | -12.62 (10.60; 19.25) | **6.76 (5.28; 18.11)** | 19.74 | 353.04 | 10.46 | <.01 | 0.96 | intercept |
| WASO (Minutes) | Bias | 0.25 (43.87) | -5.09 (40.47) | **-13.88 (34.92; 27.84)** | -13.51 (31.52; 27.73) | -5.34 | 387.59 | -2.05 | 0.041 | 0.12 | intercept |
| TST (Minutes) | Bias | 13.64 (43.49) | -1.87 (44.60) | 25.35 (37.69; 25.25) | **3.22 (37.35; 29.36)** | -17.94 | 33.08 | -5.21 | <.01 | 0.39 | slope and intercept |
| SE (%) | MAE | 6.02 (6.56) | 6.27 (6.46) | 7.19 (6.38; 4.02) | 6.66 (5.24; 4.69) | -0.07 | 21.92 | -0.13 | 0.897 | 0.01 | slope and intercept |
| SOL (Minutes) | MAE | 13.45 (22.13) | 13.43 (14.68) | 12.72 (10.53; 19.22) | 13.30 (4.49; 13.93) | -0.02 | 354.37 | -0.01 | 0.990 | 0.00 | intercept |
| WASO (Minutes) | MAE | 29.96 (31.98) | 26.01 (31.37) | 31.75 (20.64; 25.58) | 29.37 (20.67; 25.06) | -3.95 | 386.47 | -1.69 | 0.092 | 0.12 | intercept |
| TST (Minutes) | MAE | 30.23 (34.06) | 30.30 (32.73) | 35.60 (29.16; 22.58) | 32.60 (24.64; 24.34) | -0.73 | 16.58 | -0.30 | 0.770 | 0.02 | slope and intercept |

**Notes:**

TCN = Temporal convolutional network

MAE = mean absolute error

SE = Sleep efficiency

SOL = Sleep onset latency

WASO = Wake after sleep onset

TST = Total sleep time

Bolded values indicate the more favorable outcomes for comparisons that are statistically significant at an alpha level of .05.

Mixed-effects models compared the Oakley algorithm versus TCN model classification, with days nested within participants.

Grand mean values represent the percentage of predictions in that cell, averaged across recordings regardless of clustering within participants. The standard deviation of these values is represented in parentheses.

Mixed-effect mean values are derived by fitting mixed-effects models separately to each condition with a fixed-effect of intercept, and a random-effect of intercept grouped within participants. Standard deviation values are at the level of the cluster, and residual.

Difference is the condition difference estimated from the mixed-effects model used to compute inferential statistics. This value is similar but not necessarily the same as the difference between the mixed-effect condition means, which are estimated independently.

The EcoSleep dataset was excluded from comparisons of SOL or SE, as the method of imputing in-bed labels for the EcoSleep data cannot identify pre-sleep wakefulness within the in-bed interval.

Table S9. Discrepancy (CCC and CCCLON) of interval level statistics for ~24-hour and in-bed intervals, for the causal model that incorporates only the current and prior epochs.

~24-Hour Data, ~24-Hour Interval Evaluation (CCC and CCCLON), Causal Model

| Metric | Measure | Oakley | TCN | Difference | Difference Excludes 0 | Incomplete Bootstrap Samples |
| --- | --- | --- | --- | --- | --- | --- |
| TST | CCC | 0.18 [0.14, 0.22] | **0.89 [0.86, 0.91]** | -0.71 [-0.74, -0.67] | * |  |
| TST | CCCLON | 0.06 [0.01, 0.11] | **0.73 [0.61, 0.82]** | -0.67 [-0.76, -0.51] | * | 29 |

~24-Hour Data, In-Bed Interval Evaluation (CCC and CCCLON), Causal Model

| Metric | Measure | Oakley | TCN | Difference | Difference Excludes 0 | Incomplete Bootstrap Samples |
| --- | --- | --- | --- | --- | --- | --- |
| SE | CCC | 0.34 [0.23, 0.45] | **0.50 [0.39, 0.60]** | -0.16 [-0.33, -0.03] | * |  |
| SOL | CCC | 0.05 [0.02, 0.09] | **0.55 [0.45, 0.63]** | -0.49 [-0.64, -0.36] | * |  |
| WASO | CCC | 0.34 [0.23, 0.45] | **0.52 [0.42, 0.61]** | -0.18 [-0.30, -0.07] | * |  |
| TST | CCC | 0.91 [0.88, 0.93] | 0.92 [0.89, 0.93] | -0.01 [-0.03, 0.01] |  |  |
| SE | CCCLON | 0.29 [0.08, 0.48] | **0.44 [0.26, 0.60]** | -0.15 [-0.38, -0.02] | * | 1,081 |
| SOL | CCCLON | 0.07 [-0.08, 0.21] | **0.51 [0.38, 0.62]** | -0.44 [-0.55, -0.28] | * | 504 |
| WASO | CCCLON | 0.26 [0.07, 0.43] | 0.38 [0.20, 0.53] | -0.12 [-0.29, 0.03] |  | 873 |
| TST | CCCLON | 0.77 [0.66, 0.85] | 0.80 [0.69, 0.87] | -0.02 [-0.11, 0.03] |  | 0 |

**Notes:**

TCN = Temporal convolutional network

CCC = concordance correlation coefficient, calculated across days without respect to clustering within participants

CCCLON = concordance correlation coefficient with longitudinal repeated measures, calculated across days while accounting for clustering of days within participants

SE = Sleep efficiency

SOL = Sleep onset latency

WASO = Wake after sleep onset

TST = Total sleep time

Bolded values indicate the more favorable outcomes for comparisons with a 95% confidence interval of the difference excluding 0.

The CCCLON internally fits a mixed-effects model to the data to obtain variance components. Some permutations of the bootstrap can produce data sets that can’t be appropriately fit with a mixed-effects model (for example non-negative approximate variance-covariance). The column Incomplete Bootstrap Samples records the number of instances in which this occurred (out of 10,000).

The EcoSleep dataset was excluded from comparisons of SOL or SE, as the method of imputing in-bed labels for the EcoSleep data cannot identify pre-sleep wakefulness within the in-bed interval.

**MESA Performance using Causal TCN**

Epoch-level performance of the alternative model that uses only causal convolutions are presented within Table S10 for the MESA data, at both all-available and in-bed intervals. At the all-available interval, the causal TCN produced more favorable epoch-by-epoch performance relative to the Oakley classifier on balanced accuracy, specificity, PPV, and MCC, while the Oakley classifier performance more favorably on sensitivity, NPV, and F1-Score. At the in-bed interval, the causal TCN produced more favorable epoch-by-epoch performance on balanced accuracy, specificity, and PPV, while the Oakley classifier produced more favorable epoch-by-epoch performance on accuracy, sensitivity, NPV, F1-Score, MCC, and PABAK.

Discrepancy of the causal TCN within the all-available and in-bed intervals are presented within Table S11 for bias and MAE, and Table S12 for CCC. The causal TCN classifier failed to capture any sleep time for 6 of the 1,690 records evaluated. With no sleep onset, SOL and WASO are not defined for those records for the TCN classifier. To compare on the same data, those 6 records were also not analyzed for SOL or WASO for the Oakley classifier. Across the all-available interval, the causal TCN produced more favorable bias, MAE, and CCC of TST. Across the in-bed interval, the causal TCN produced more favorable bias and CCC of SE, CCC of SOL, bias and CCC of WASO, and bias of TST. The Oakley classifier produced more favorable bias and MAE of SOL, and MAE of WASO.

Table S10. Epoch-level classification performance on the Multi-Ethnic Study of Atherosclerosis (MESA) data set, for All-Available and In-Bed periods, for the causal model that only uses the current and prior epochs.

MESA Data, All-Available Interval Epoch-by-Epoch Evaluation, Causal Model

| Metric | Oakley (Grand) | TCN (Grand) | Difference | DF | t | p | d | Random Effect Structure |
| --- | --- | --- | --- | --- | --- | --- | --- | --- |
| AUC |  | 0.847 (0.119) |  |  |  |  |  |  |
| Accuracy | 0.791 (0.102) | 0.791 (0.127) | 0.000 | 1,689 | 0.03 | 0.977 | 0.00 | intercept |
| Balanced Accuracy | 0.694 (0.091) | **0.759 (0.116)** | 0.065 | 1,689 | 27.67 | <.01 | 0.63 | intercept |
| Sensitivity | **0.960 (0.047)** | 0.812 (0.205) | -0.148 | 1,689 | -33.05 | <.01 | 1.00 | intercept |
| Specificity | 0.428 (0.177) | **0.706 (0.214)** | 0.278 | 1,689 | 65.79 | <.01 | 1.42 | intercept |
| PPV | 0.780 (0.126) | **0.861 (0.126)** | 0.081 | 1,689 | 35.21 | <.01 | 0.65 | intercept |
| NPV | **0.821 (0.181)** | 0.686 (0.216) | -0.134 | 1,689 | -33.32 | <.01 | 0.67 | intercept |
| F1-Score | **0.854 (0.091)** | 0.817 (0.165) | -0.037 | 1,689 | -11.13 | <.01 | 0.28 | intercept |
| MCC | 0.476 (0.180) | **0.528 (0.226)** | 0.052 | 1,689 | 12.05 | <.01 | 0.26 | intercept |
| PABAK | 0.582 (0.204) | 0.582 (0.254) | 0.000 | 1,689 | 0.03 | 0.977 | 0.00 | intercept |

MESA Data, In-Bed Interval Epoch-by-Epoch Evaluation, Causal Model

| Metric | Oakley (Grand) | TCN (Grand) | Difference | DF | t | p | d | Random Effect Structure |
| --- | --- | --- | --- | --- | --- | --- | --- | --- |
| AUC |  | 0.798 (0.133) |  |  |  |  |  |  |
| Accuracy | **0.820 (0.104)** | 0.776 (0.138) | -0.044 | 1,689 | -14.51 | <.01 | 0.36 | intercept |
| Balanced Accuracy | 0.673 (0.094) | **0.703 (0.118)** | 0.030 | 1,689 | 12.69 | <.01 | 0.28 | intercept |
| Sensitivity | **0.960 (0.047)** | 0.812 (0.205) | -0.148 | 1,689 | -33.04 | <.01 | 0.99 | intercept |
| Specificity | 0.387 (0.184) | **0.594 (0.253)** | 0.207 | 1,689 | 42.00 | <.01 | 0.94 | intercept |
| PPV | 0.826 (0.123) | **0.870 (0.122)** | 0.044 | 1,689 | 20.51 | <.01 | 0.36 | intercept |
| NPV | **0.741 (0.213)** | 0.563 (0.242) | -0.178 | 1,689 | -37.99 | <.01 | 0.78 | intercept |
| F1-Score | **0.882 (0.086)** | 0.821 (0.165) | -0.061 | 1,689 | -18.39 | <.01 | 0.46 | intercept |
| MCC | **0.434 (0.187)** | 0.411 (0.227) | -0.023 | 1,689 | -5.25 | <.01 | 0.11 | intercept |
| PABAK | **0.640 (0.209)** | 0.552 (0.276) | -0.088 | 1,689 | -14.51 | <.01 | 0.36 | intercept |

**Notes:**

TCN = Temporal convolutional network

AUC = Area under the receiver operating characteristic curve

PABAK = Prevalence-adjusted and bias-adjusted kappa

PPV = Positive predictive value

NPV = Negative predictive value

MCC = Matthews correlation coefficient

TCN = Temporal Convolution Network

DF = Mixed-effects model degrees of freedom

t = Mixed-effects model t-value for the comparison between classifiers

Bolded values indicate the more favorable outcomes for comparisons that are statistically significant at an alpha level of .05.

Grand mean values are averaged across recordings (MESA data was not nested). The standard deviation of these values is represented in parentheses.

Table S11. Discrepancy (Bias and MAE) of interval level statistics for All-Available and In-Bed intervals on the Multi-Ethnic Study of Atherosclerosis (MESA) data set, for the causal model that only uses the current and prior epochs.

MESA All-Available Interval Evaluation (Bias and MAE), Causal Model

| Metric | Measure | Oakley (Grand) | TCN (Grand) | Difference | DF | t | p | d | Random Effect Structure |
| --- | --- | --- | --- | --- | --- | --- | --- | --- | --- |
| TST (Minutes) | Bias | 83.85 (61.91) | **-16.89 (88.99)** | -100.74 | 1,689 | -49.47 | <.01 | 1.31 | intercept |
| TST (Minutes) | MAE | 85.22 (60.01) | **62.32 (65.72)** | -22.90 | 1,689 | -11.60 | <.01 | 0.36 | intercept |

MESA In-Bed Interval Evaluation (Bias and MAE), Causal Model

| Metric | Measure | Oakley (Grand) | TCN (Grand) | Difference | DF | t | p | d | Random Effect Structure |
| --- | --- | --- | --- | --- | --- | --- | --- | --- | --- |
| SE (%) | Bias | 12.07 (10.98) | **-4.64 (18.34)** | -16.71 | 1,689 | -40.98 | <.01 | 1.11 | intercept |
| SOL (Minutes) | Bias | **-19.29 (35.56)** | 27.68 (56.92) | 46.97 | 1,683 | 33.82 | <.01 | 0.99 | intercept |
| WASO (Minutes) | Bias | -40.18 (55.56) | **-13.33 (75.89)** | 26.85 | 1,683 | 16.18 | <.01 | 0.40 | intercept |
| TST (Minutes) | Bias | 58.91 (55.86) | **-20.42 (87.13)** | -79.33 | 1,689 | -40.20 | <.01 | 1.08 | intercept |
| SE (%) | MAE | 12.69 (10.26) | 12.98 (13.76) | 0.29 | 1,689 | 0.74 | 0.460 | 0.02 | intercept |
| SOL (Minutes) | MAE | **19.99 (35.17)** | 36.59 (51.64) | 16.60 | 1,683 | 11.37 | <.01 | 0.38 | intercept |
| WASO (Minutes) | MAE | **49.18 (47.77)** | 54.37 (54.58) | 5.19 | 1,683 | 3.94 | <.01 | 0.10 | intercept |
| TST (Minutes) | MAE | 61.62 (52.85) | 61.17 (65.31) | -0.45 | 1,689 | -0.25 | 0.806 | 0.01 | intercept |

**Notes:**

TCN = Temporal convolutional network

MAE = mean absolute error

SE = Sleep efficiency

SOL = Sleep onset latency

WASO = Wake after sleep onset

TST = Total sleep time

Grand mean values are averaged across recordings (MESA data was not clustered). The standard deviation of these values is represented in parentheses.

Bolded values indicate the more favorable outcomes for comparisons that are statistically significant at an alpha level of .05.

Table S12. Discrepancy (CCC) of interval level statistics for All-Available and In-Bed intervals on the Multi-Ethnic Study of Atherosclerosis (MESA) data set, for the causal model that only uses the current and prior epochs.

MESA All-Available Interval Evaluation (CCC), Causal Model

| Metric | Measure | Oakley | TCN | Difference | Difference Excludes 0 |
| --- | --- | --- | --- | --- | --- |
| TST | CCC | 0.45 [0.43, 0.48] | **0.59 [0.56, 0.62]** | -0.14 [-0.17, -0.10] | * |

MESA In-Bed Interval Evaluation (CCC), Causal Model

| Metric | Measure | Oakley | TCN | Difference | Difference Excludes 0 |
| --- | --- | --- | --- | --- | --- |
| SE | CCC | 0.29 [0.27, 0.32] | **0.44 [0.41, 0.48]** | -0.15 [-0.19, -0.11] | * |
| SOL | CCC | 0.07 [0.06, 0.08] | **0.26 [0.23, 0.29]** | -0.19 [-0.25, -0.13] | * |
| WASO | CCC | 0.33 [0.30, 0.36] | **0.45 [0.41, 0.49]** | -0.12 [-0.17, -0.07] | * |
| TST | CCC | 0.59 [0.57, 0.62] | 0.60 [0.57, 0.63] | -0.01 [-0.05, 0.03] |  |

**Notes:**

TCN = Temporal convolutional network

CCC = concordance correlation coefficient

SE = Sleep efficiency

SOL = Sleep onset latency

WASO = Wake after sleep onset

TST = Total sleep time

Bolded values indicate the more favorable outcomes for comparisons with a 95% confidence interval of the difference excluding 0.

**2c. Bidirectional Model Performance Relative to Alternative Classifiers**

**Comparison to Sadeh Classifier on ~24-Hour Dataset**

Epoch-by-epoch classification performance in comparing the (bidirectional) TCN classifier to the Sadeh classifier is presented within Table S13 for the ~24-Hour data, at both ~24-Hour and in-bed intervals. At the ~24 Hour interval, the TCN produces favorable epoch-by-epoch performance relative to the Sadeh classifier on accuracy, balanced accuracy, specificity, PPV, F1-Score, MCC, and PABAK. The Sadeh classifier produced favorable epoch-by-epoch performance on sensitivity and NPV. Within the in-bed interval, the TCN produced favorable epoch-by-epoch performance relative to the Sadeh classifier on balanced accuracy, specificity, PPV, and MCC. The Sadeh classifier produced favorable epoch-by-epoch performance on sensitivity, while accuracy, NPV, F1-Score, and PABAK did not differ.

Discrepancy of the TCN and Sadeh classifiers within the ~24-Hour and in-bed intervals are presented within Table S14 for bias and MAE, and Table S15 for CCC/CCCLON. At the ~24-Hour interval, the TCN has favorable estimates of TST in terms of bias, MAE, and both CCC and CCCLON. At the in-bed interval, the TCN has favorable performance in terms of bias, CCC, and CCCLON of SE, bias, MAE, CCC, and CCCLON of SOL, bias and CCC of WASO, and bias, MAE, and CCC of TST.

Table S13. Epoch-level classification performance for both the ~24-hour and in-bed intervals for both the Sadeh and TCN classifiers.

~24-Hour Interval Epoch-by-Epoch Evaluation, Sadeh vs. TCN

| Metric | Sadeh (Grand) | TCN (Grand) | Sadeh (Mixed) | TCN (Mixed) | Difference | DF | t | p | d | Random Effect Structure |
| --- | --- | --- | --- | --- | --- | --- | --- | --- | --- | --- |
| AUC |  | 0.993 (0.011) |  | 0.989 (0.012; 0.007) |  |  |  |  |  |  |
| Accuracy | 0.728 (0.103) | 0.963 (0.035) | 0.714 (0.075; 0.073) | **0.955 (0.026; 0.026)** | 0.235 | 391.85 | 37.24 | <.01 | 3.05 | intercept |
| Balanced Accuracy | 0.799 (0.074) | 0.960 (0.038) | 0.788 (0.052; 0.055) | **0.952 (0.032; 0.028)** | 0.169 | 45.93 | 17.66 | <.01 | 3.11 | slope and intercept |
| Sensitivity | 0.982 (0.024) | 0.954 (0.069) | **0.983 (0.019; 0.015)** | 0.946 (0.062; 0.047) | -0.036 | 35.92 | -4.42 | <.01 | 0.52 | slope and intercept |
| Specificity | 0.615 (0.153) | 0.967 (0.041) | 0.593 (0.111; 0.108) | **0.958 (0.029; 0.032)** | 0.352 | 392.73 | 38.52 | <.01 | 3.13 | intercept |
| PPV | 0.538 (0.125) | 0.934 (0.082) | 0.529 (0.070; 0.105) | **0.912 (0.067; 0.055)** | 0.393 | 40.92 | 32.93 | <.01 | 3.78 | slope and intercept |
| NPV | 0.988 (0.017) | 0.981 (0.029) | **0.988 (0.011; 0.013)** | 0.977 (0.024; 0.021) | -0.010 | 30.50 | -3.27 | <.01 | 0.34 | slope and intercept |
| F1-Score | 0.686 (0.102) | 0.940 (0.059) | 0.680 (0.057; 0.086) | **0.925 (0.050; 0.041)** | 0.255 | 42.43 | 24.09 | <.01 | 3.16 | slope and intercept |
| MCC | 0.557 (0.125) | 0.917 (0.076) | 0.543 (0.085; 0.095) | **0.896 (0.063; 0.055)** | 0.361 | 42.81 | 23.48 | <.01 | 3.63 | slope and intercept |
| PABAK | 0.456 (0.206) | 0.927 (0.069) | 0.428 (0.151; 0.146) | **0.910 (0.051; 0.052)** | 0.471 | 391.85 | 37.24 | <.01 | 3.05 | intercept |

In-Bed Interval Epoch-by-Epoch Evaluation, Sadeh vs. TCN

| Metric | Sadeh (Grand) | TCN (Grand) | Sadeh (Mixed) | TCN (Mixed) | Difference | DF | t | p | d | Random Effect Structure |
| --- | --- | --- | --- | --- | --- | --- | --- | --- | --- | --- |
| AUC |  | 0.925 (0.071) |  | 0.903 (0.062; 0.047) |  |  |  |  |  |  |
| Accuracy | 0.906 (0.079) | 0.911 (0.080) | 0.887 (0.067; 0.053) | 0.889 (0.071; 0.051) | 0.004 | 45.31 | 0.75 | 0.459 | 0.04 | slope and intercept |
| Balanced Accuracy | 0.675 (0.108) | 0.773 (0.124) | 0.653 (0.076; 0.073) | **0.738 (0.091; 0.083)** | 0.091 | 45.41 | 10.99 | <.01 | 0.79 | slope and intercept |
| Sensitivity | 0.982 (0.024) | 0.956 (0.067) | **0.983 (0.018; 0.015)** | 0.951 (0.056; 0.047) | -0.031 | 35.50 | -4.26 | <.01 | 0.49 | slope and intercept |
| Specificity | 0.368 (0.226) | 0.589 (0.265) | 0.321 (0.160; 0.148) | **0.524 (0.197; 0.175)** | 0.209 | 44.06 | 11.70 | <.01 | 0.85 | slope and intercept |
| PPV | 0.916 (0.084) | 0.942 (0.073) | 0.895 (0.072; 0.056) | **0.919 (0.069; 0.042)** | 0.025 | 386.22 | 5.59 | <.01 | 0.29 | intercept |
| NPV | 0.699 (0.231) | 0.679 (0.225) | 0.719 (0.129; 0.195) | 0.695 (0.146; 0.172) | -0.020 | 32.93 | -0.80 | 0.432 | 0.09 | slope and intercept |
| F1-Score | 0.946 (0.051) | 0.946 (0.054) | 0.934 (0.044; 0.034) | 0.931 (0.051; 0.035) | 0.000 | 384.77 | 0.02 | 0.986 | 0.00 | intercept |
| MCC | 0.436 (0.180) | 0.554 (0.182) | 0.410 (0.130; 0.128) | **0.515 (0.130; 0.126)** | 0.116 | 29.17 | 8.08 | <.01 | 0.64 | slope and intercept |
| PABAK | 0.812 (0.158) | 0.821 (0.160) | 0.775 (0.135; 0.106) | 0.779 (0.143; 0.103) | 0.007 | 45.46 | 0.75 | 0.459 | 0.04 | slope and intercept |

**Notes:**

TCN = Temporal convolutional network

AUC = Area under the receiver operating characteristic curve

PABAK = Prevalence-adjusted and bias-adjusted kappa

PPV = Positive predictive value

NPV = Negative predictive value

MCC = Matthews correlation coefficient

TCN = Temporal Convolution Network

DF = Mixed-effects model degrees of freedom

t = Mixed-effects model t-value for the comparison between classifiers

Bolded values indicate the more favorable outcomes for comparisons that are statistically significant at an alpha level of .05.

AUC not possible for Sadeh classifier given discrete classification.

Mixed-effects models compared the manufacturer’s standard algorithm versus TCN model classification, with days nested within participants.

Grand mean values are averaged across recordings regardless of clustering within participants. The standard deviation of these values is represented in parentheses.

Mixed-effect mean values are derived by fitting mixed-effects models separately to each condition with a fixed-effect of intercept, and a random-effect of intercept grouped within participants. Standard deviation values are at the level of the cluster, and residual.

Difference is the condition difference estimated from the mixed-effects model used to compute inferential statistics. This value is similar but not necessarily the same as the difference between the mixed-effect condition means, which are estimated independently.

For EcoSleep Study, in-bed interval was not set for participants, so nighttime sleep interval imputed from PSG scored start/end of nighttime sleep epochs.

Table S14. Discrepancy (bias and MAE) of interval level statistics for ~24-hour and in-bed intervals for Sadeh and TCN Classifiers.

~24-Hour Interval Evaluation (Bias and MAE) for Sadeh and TCN

| Metric | Measure | Sadeh (Grand) | TCN (Grand) | Sadeh (Mixed) | TCN (Mixed) | Difference | DF | t | p | d | Random Effect Structure |
| --- | --- | --- | --- | --- | --- | --- | --- | --- | --- | --- | --- |
| TST (Minutes) | Bias | 358.94 (153.12) | 12.82 (52.36) | 374.48 (118.82; 102.37) | **18.56 (40.79; 36.72)** | -346.12 | 389.39 | -39.30 | <.01 | 3.00 | intercept |
| TST (Minutes) | MAE | 358.94 (153.12) | 35.22 (40.76) | 374.48 (118.82; 102.37) | **39.98 (25.24; 33.28)** | -323.73 | 390.14 | -35.59 | <.01 | 2.86 | intercept |

In-Bed Interval Evaluation (Bias and MAE) for Sadeh and TCN

| Metric | Measure | Sadeh (Grand) | TCN (Grand) | Sadeh (Mixed) | TCN (Mixed) | Difference | DF | t | p | d | Random Effect Structure |
| --- | --- | --- | --- | --- | --- | --- | --- | --- | --- | --- | --- |
| SE (%) | Bias | 5.83 (8.55) | 1.41 (9.10) | 7.99 (7.94; 5.25) | **3.56 (8.08; 5.67)** | -4.32 | 33.65 | -6.21 | <.01 | 0.44 | slope and intercept |
| SOL (Minutes) | Bias | -12.89 (22.27) | -0.45 (12.64) | -12.22 (10.60; 19.35) | **-0.87 (5.10; 11.76)** | 12.44 | 348.38 | 7.29 | <.01 | 0.68 | intercept |
| WASO (Minutes) | Bias | -19.41 (41.10) | -13.91 (40.36) | -31.58 (32.02; 27.59) | **-23.62 (33.90; 26.79)** | 5.50 | 385.96 | 2.15 | 0.032 | 0.13 | intercept |
| TST (Minutes) | Bias | 32.98 (43.60) | 8.97 (45.97) | 42.59 (35.11; 29.04) | **15.62 (39.31; 29.42)** | -24.40 | 36.19 | -6.73 | <.01 | 0.51 | slope and intercept |
| SE (%) | MAE | 6.67 (7.91) | 5.97 (7.01) | 8.54 (7.41; 5.05) | 6.96 (5.91; 4.90) | -1.26 | 30.96 | -1.79 | 0.084 | 0.15 | slope and intercept |
| SOL (Minutes) | MAE | 13.20 (22.09) | 6.92 (10.58) | 12.47 (10.83; 18.98) | **7.09 (5.10; 9.41)** | -5.68 | 41.92 | -2.56 | 0.014 | 0.35 | slope and intercept |
| WASO (Minutes) | MAE | 29.95 (34.16) | 26.42 (33.50) | 37.21 (26.17; 25.07) | 33.17 (26.36; 24.34) | -3.53 | 385.30 | -1.52 | 0.129 | 0.10 | intercept |
| TST (Minutes) | MAE | 36.07 (41.07) | 30.58 (35.43) | 44.30 (33.11; 28.32) | **35.46 (26.69; 25.91)** | -7.68 | 47.27 | -2.48 | 0.017 | 0.19 | slope and intercept |

**Notes:**

TCN = Temporal convolutional network

MAE = mean absolute error

SE = Sleep efficiency

SOL = Sleep onset latency

WASO = Wake after sleep onset

TST = Total sleep time

Bolded values indicate the more favorable outcomes for comparisons that are statistically significant at an alpha level of .05.

Mixed-effects models compared the Sadeh algorithm versus TCN model classification, with days nested within participants.

Grand mean values represent the percentage of predictions in that cell, averaged across recordings regardless of clustering within participants. The standard deviation of these values is represented in parentheses.

Mixed-effect mean values are derived by fitting mixed-effects models separately to each condition with a fixed-effect of intercept, and a random-effect of intercept grouped within participants. Standard deviation values are at the level of the cluster, and residual.

Difference is the condition difference estimated from the mixed-effects model used to compute inferential statistics. This value is similar but not necessarily the same as the difference between the mixed-effect condition means, which are estimated independently.

The EcoSleep dataset was excluded from comparisons of SOL or SE, as the method of imputing in-bed labels for the EcoSleep data cannot identify pre-sleep wakefulness within the in-bed interval.

Table S15. Discrepancy (CCC and CCCLON) of interval level statistics for ~24-hour and In-Bed intervals for Sadeh and TCN Classifiers.

~24-Hour Interval Evaluation (CCC and CCCLON) for Sadeh and TCN

| Metric | Measure | Sadeh | TCN | Difference | Difference Excludes 0 | Incomplete Bootstrap Samples |
| --- | --- | --- | --- | --- | --- | --- |
| TST | CCC | 0.12 [0.09, 0.15] | **0.88 [0.85, 0.91]** | -0.77 [-0.80, -0.73] | * |  |
| TST | CCCLON | 0.05 [0.00, 0.09] | **0.71 [0.59, 0.81]** | -0.67 [-0.77, -0.49] | * | 38 |

In-Bed Interval Evaluation (CCC and CCCLON) for Sadeh and TCN

| Metric | Measure | Sadeh | TCN | Difference | Difference Excludes 0 | Incomplete Bootstrap Samples |
| --- | --- | --- | --- | --- | --- | --- |
| SE | CCC | 0.24 [0.16, 0.32] | **0.50 [0.38, 0.59]** | -0.26 [-0.39, -0.15] | * |  |
| SOL | CCC | 0.05 [0.01, 0.09] | **0.84 [0.79, 0.88]** | -0.79 [-0.89, -0.66] | * |  |
| WASO | CCC | 0.28 [0.19, 0.37] | **0.46 [0.36, 0.55]** | -0.18 [-0.33, -0.06] | * |  |
| TST | CCC | 0.88 [0.85, 0.91] | **0.91 [0.88, 0.93]** | -0.03 [-0.06, -0.01] | * |  |
| SE | CCCLON | 0.22 [0.06, 0.36] | **0.41 [0.22, 0.57]** | -0.19 [-0.35, -0.04] | * | 834 |
| SOL | CCCLON | 0.07 [-0.08, 0.22] | **0.82 [0.75, 0.87]** | -0.75 [-0.91, -0.53] | * | 1,779 |
| WASO | CCCLON | 0.26 [0.10, 0.40] | 0.29 [0.13, 0.44] | -0.03 [-0.21, 0.18] |  | 2,020 |
| TST | CCCLON | 0.74 [0.63, 0.82] | 0.76 [0.65, 0.84] | -0.02 [-0.08, 0.09] |  | 0 |

**Notes:**

TCN = Temporal convolutional network

CCC = concordance correlation coefficient, calculated across days without respect to clustering within participants

CCCLON = concordance correlation coefficient with longitudinal repeated measures, calculated across days while accounting for clustering of days within participants

SE = Sleep efficiency

SOL = Sleep onset latency

WASO = Wake after sleep onset

TST = Total sleep time

Bolded values indicate the more favorable outcomes for comparisons with a 95% confidence interval of the difference excluding 0.

The CCCLON internally fits a mixed-effects model to the data to obtain variance components. Some permutations of the bootstrap can produce data sets that can’t be appropriately fit with a mixed-effects model (for example non-negative approximate variance-covariance). The column Incomplete Bootstrap Samples records the number of instances in which this occurred (out of 10,000).

The EcoSleep dataset was excluded from comparisons of SOL or SE, as the method of imputing in-bed labels for the EcoSleep data cannot identify pre-sleep wakefulness within the in-bed interval.

**Comparison to Scripps Clinic Classifier on ~24-Hour Dataset**

Epoch-by-epoch classification performance in comparing the (bidirectional) TCN classifier to the Scripps Clinic classifier is presented within Table S16 for the ~24-Hour data, at both ~24-Hour and in-bed intervals. At the ~24 Hour interval, the TCN produces favorable epoch-by-epoch performance relative to the Scripps Clinic classifier on accuracy, balanced accuracy, specificity, PPV, F1-Score, MCC, and PABAK. The Scripps Clinic classifier produced favorable sensitivity, while NPV did not significantly differ. Within the in-bed interval, the TCN produced favorable epoch-by-epoch performance relative to the Scripps Clinic classifier on balanced accuracy, specificity, PPV, NPV, and MCC. Accuracy, sensitivity, F1-Score, and PABAK did not significantly differ.

Discrepancy of the TCN and Scripps Clinic classifiers within the ~24-Hour and in-bed intervals are presented within Table S17 for bias and MAE, and Table S18 for CCC/CCCLON. At the ~24-Hour interval, the TCN has favorable estimates of TST in terms of bias, MAE, and both CCC and CCCLON. At the in-bed interval, the TCN has favorable performance in terms of bias and CCC of SE, bias, MAE, and CCC/CCCLON of SOL, and bias in TST, while the Scripps Clinic classifier has favorable performance in terms of bias of WASO.

Table S16. Epoch-level classification performance for both the ~24-hour and In-Bed intervals for the Scripps Clinic and TCN classifiers.

~24-Hour Interval Epoch-by-Epoch Evaluation, Scripps Clinic vs. TCN

| Metric | Scripps Clinic (Grand) | TCN (Grand) | Scripps Clinic (Mixed) | TCN (Mixed) | Difference | DF | t | p | d | Random Effect Structure |
| --- | --- | --- | --- | --- | --- | --- | --- | --- | --- | --- |
| AUC |  | 0.993 (0.011) |  | 0.989 (0.012; 0.007) |  |  |  |  |  |  |
| Accuracy | 0.789 (0.094) | 0.963 (0.035) | 0.777 (0.064; 0.072) | **0.955 (0.026; 0.026)** | 0.174 | 391.29 | 29.42 | <.01 | 2.43 | intercept |
| Balanced Accuracy | 0.837 (0.068) | 0.960 (0.038) | 0.830 (0.041; 0.056) | **0.952 (0.032; 0.028)** | 0.128 | 41.90 | 15.72 | <.01 | 2.44 | slope and intercept |
| Sensitivity | 0.963 (0.038) | 0.954 (0.069) | **0.966 (0.031; 0.020)** | 0.946 (0.062; 0.047) | -0.019 | 35.55 | -2.27 | 0.030 | 0.28 | slope and intercept |
| Specificity | 0.711 (0.143) | 0.967 (0.041) | 0.692 (0.096; 0.109) | **0.958 (0.029; 0.032)** | 0.255 | 393.03 | 29.25 | <.01 | 2.42 | intercept |
| PPV | 0.610 (0.134) | 0.934 (0.082) | 0.598 (0.080; 0.108) | **0.912 (0.067; 0.055)** | 0.326 | 41.95 | 25.77 | <.01 | 3.03 | slope and intercept |
| NPV | 0.977 (0.025) | 0.981 (0.029) | 0.979 (0.015; 0.019) | 0.977 (0.024; 0.021) | 0.000 | 28.70 | 0.04 | 0.971 | 0.00 | slope and intercept |
| F1-Score | 0.737 (0.098) | 0.940 (0.059) | 0.730 (0.058; 0.081) | **0.925 (0.050; 0.041)** | 0.204 | 41.11 | 20.14 | <.01 | 2.60 | slope and intercept |
| MCC | 0.627 (0.127) | 0.917 (0.076) | 0.615 (0.079; 0.102) | **0.896 (0.063; 0.055)** | 0.292 | 40.65 | 20.45 | <.01 | 2.90 | slope and intercept |
| PABAK | 0.579 (0.188) | 0.927 (0.069) | 0.555 (0.127; 0.143) | **0.910 (0.051; 0.052)** | 0.348 | 391.29 | 29.42 | <.01 | 2.43 | intercept |

In-Bed Interval Epoch-by-Epoch Evaluation, Scripps Clinic vs. TCN

| Metric | Scripps Clinic (Grand) | TCN (Grand) | Scripps Clinic (Mixed) | TCN (Mixed) | Difference | DF | t | p | d | Random Effect Structure |
| --- | --- | --- | --- | --- | --- | --- | --- | --- | --- | --- |
| AUC |  | 0.925 (0.071) |  | 0.903 (0.062; 0.047) |  |  |  |  |  |  |
| Accuracy | 0.903 (0.073) | 0.911 (0.080) | 0.888 (0.063; 0.048) | 0.889 (0.071; 0.051) | 0.007 | 385.27 | 1.55 | 0.122 | 0.09 | intercept |
| Balanced Accuracy | 0.749 (0.127) | 0.773 (0.124) | 0.716 (0.092; 0.086) | **0.738 (0.091; 0.083)** | 0.024 | 390.58 | 3.00 | <.01 | 0.19 | intercept |
| Sensitivity | 0.963 (0.038) | 0.956 (0.067) | 0.966 (0.030; 0.020) | 0.951 (0.056; 0.047) | -0.015 | 34.74 | -1.90 | 0.065 | 0.23 | slope and intercept |
| Specificity | 0.534 (0.270) | 0.589 (0.265) | 0.464 (0.202; 0.170) | **0.524 (0.197; 0.175)** | 0.059 | 41.46 | 3.20 | <.01 | 0.23 | slope and intercept |
| PPV | 0.930 (0.081) | 0.942 (0.073) | 0.907 (0.072; 0.051) | **0.919 (0.069; 0.042)** | 0.012 | 386.26 | 2.81 | <.01 | 0.14 | intercept |
| NPV | 0.616 (0.208) | 0.679 (0.225) | 0.653 (0.141; 0.156) | **0.695 (0.146; 0.172)** | 0.054 | 40.02 | 2.36 | 0.023 | 0.25 | slope and intercept |
| F1-Score | 0.943 (0.048) | 0.946 (0.054) | 0.933 (0.042; 0.032) | 0.931 (0.051; 0.035) | 0.002 | 384.66 | 0.79 | 0.429 | 0.04 | intercept |
| MCC | 0.490 (0.164) | 0.554 (0.182) | 0.466 (0.111; 0.125) | **0.515 (0.130; 0.126)** | 0.064 | 390.40 | 5.24 | <.01 | 0.37 | intercept |
| PABAK | 0.807 (0.146) | 0.821 (0.160) | 0.775 (0.125; 0.097) | 0.779 (0.143; 0.103) | 0.015 | 385.27 | 1.55 | 0.122 | 0.09 | intercept |

**Notes:**

TCN = Temporal convolutional network

AUC = Area under the receiver operating characteristic curve

PABAK = Prevalence-adjusted and bias-adjusted kappa

PPV = Positive predictive value

NPV = Negative predictive value

MCC = Matthews correlation coefficient

TCN = Temporal Convolution Network

DF = Mixed-effects model degrees of freedom

t = Mixed-effects model t-value for the comparison between classifiers

Bolded values indicate the more favorable outcomes for comparisons that are statistically significant at an alpha level of .05.

AUC not possible for Scripps Clinic classifier given discrete classification.

Mixed-effects models compared the manufacturer’s standard algorithm versus TCN model classification, with days nested within participants.

Grand mean values are averaged across recordings regardless of clustering within participants. The standard deviation of these values is represented in parentheses.

Mixed-effect mean values are derived by fitting mixed-effects models separately to each condition with a fixed-effect of intercept, and a random-effect of intercept grouped within participants. Standard deviation values are at the level of the cluster, and residual.

Difference is the condition difference estimated from the mixed-effects model used to compute inferential statistics. This value is similar but not necessarily the same as the difference between the mixed-effect condition means, which are estimated independently.

For EcoSleep Study, in-bed interval was not set for participants, so nighttime sleep interval imputed from PSG scored start/end of nighttime sleep epochs.

Table S17. Discrepancy (bias and MAE) of interval level statistics for ~24-hour and in-bed intervals for Scripps Clinic and TCN Classifiers.

~24-Hour Interval Evaluation (Bias and MAE) for Scripps Clinic and TCN

| Metric | Measure | Scripps Clinic (Grand) | TCN (Grand) | Scripps Clinic (Mixed) | TCN (Mixed) | Difference | DF | t | p | d | Random Effect Structure |
| --- | --- | --- | --- | --- | --- | --- | --- | --- | --- | --- | --- |
| TST (Minutes) | Bias | 257.85 (142.95) | 12.82 (52.36) | 274.66 (106.33; 98.66) | **18.56 (40.79; 36.72)** | -245.02 | 390.30 | -29.51 | <.01 | 2.27 | intercept |
| TST (Minutes) | MAE | 259.20 (140.47) | 35.22 (40.76) | 275.40 (104.85; 97.62) | **39.98 (25.24; 33.28)** | -223.98 | 390.64 | -26.56 | <.01 | 2.14 | intercept |

In-Bed Interval Evaluation (Bias and MAE) for Scripps Clinic and TCN

| Metric | Measure | Scripps Clinic (Grand) | TCN (Grand) | Scripps Clinic (Mixed) | TCN (Mixed) | Difference | DF | t | p | d | Random Effect Structure |
| --- | --- | --- | --- | --- | --- | --- | --- | --- | --- | --- | --- |
| SE (%) | Bias | 2.61 (9.02) | 1.41 (9.10) | 5.13 (8.67; 4.81) | **3.56 (8.08; 5.67)** | -1.38 | 32.56 | -2.04 | 0.049 | 0.14 | slope and intercept |
| SOL (Minutes) | Bias | -9.89 (21.09) | -0.45 (12.64) | -9.66 (10.58; 18.05) | **-0.87 (5.10; 11.76)** | 9.44 | 347.40 | 5.82 | <.01 | 0.54 | intercept |
| WASO (Minutes) | Bias | -6.61 (42.66) | -13.91 (40.36) | **-19.33 (33.22; 27.41)** | -23.62 (33.90; 26.79) | -6.21 | 264.45 | -2.38 | 0.018 | 0.15 | slope and intercept |
| TST (Minutes) | Bias | 17.42 (44.67) | 8.97 (45.97) | 28.46 (37.80; 26.02) | **15.62 (39.31; 29.42)** | -9.94 | 32.09 | -2.77 | <.01 | 0.21 | slope and intercept |
| SE (%) | MAE | 6.23 (7.01) | 5.97 (7.01) | 7.59 (6.72; 4.32) | 6.96 (5.91; 4.90) | -0.26 | 348.94 | -0.56 | 0.573 | 0.03 | intercept |
| SOL (Minutes) | MAE | 11.46 (20.28) | 6.92 (10.58) | 11.09 (10.23; 17.28) | **7.09 (5.10; 9.41)** | -4.28 | 40.84 | -2.07 | 0.044 | 0.28 | slope and intercept |
| WASO (Minutes) | MAE | 28.24 (32.61) | 26.42 (33.50) | 31.58 (21.73; 25.70) | 33.17 (26.36; 24.34) | -1.82 | 385.14 | -0.77 | 0.439 | 0.05 | intercept |
| TST (Minutes) | MAE | 31.52 (36.08) | 30.58 (35.43) | 36.90 (30.01; 24.26) | 35.46 (26.69; 25.91) | -0.94 | 387.83 | -0.40 | 0.688 | 0.02 | intercept |

**Notes:**

TCN = Temporal convolutional network

MAE = Mean absolute error

SE = Sleep efficiency

SOL = Sleep onset latency

WASO = Wake after sleep onset

TST = Total sleep time

Bolded values indicate the more favorable outcomes for comparisons that are statistically significant at an alpha level of .05.

Mixed-effects models compared the Scripps Clinic algorithm versus TCN model classification, with days nested within participants.

Grand mean values represent the percentage of predictions in that cell, averaged across recordings regardless of clustering within participants. The standard deviation of these values is represented in parentheses.

Mixed-effect mean values are derived by fitting mixed-effects models separately to each condition with a fixed-effect of intercept, and a random-effect of intercept grouped within participants. Standard deviation values are at the level of the cluster, and residual.

Difference is the condition difference estimated from the mixed-effects model used to compute inferential statistics. This value is similar but not necessarily the same as the difference between the mixed-effect condition means, which are estimated independently.

The EcoSleep dataset was excluded from comparisons of SOL or SE, as the method of imputing in-bed labels for the EcoSleep data cannot identify pre-sleep wakefulness within the in-bed interval.

Table S18. Discrepancy (CCC and CCCLON) of interval level statistics for ~24-hour and In-Bed intervals for Scripps Clinic and TCN Classifiers.

~24-Hour Interval Evaluation (CCC and CCCLON) for Scripps Clinic and TCN

| Metric | Measure | Scripps Clinic | TCN | Difference | Difference Excludes 0 | Incomplete Bootstrap Samples |
| --- | --- | --- | --- | --- | --- | --- |
| TST | CCC | 0.20 [0.15, 0.25] | **0.88 [0.85, 0.91]** | -0.68 [-0.72, -0.64] | * |  |
| TST | CCCLON | 0.07 [0.01, 0.13] | **0.71 [0.59, 0.81]** | -0.64 [-0.74, -0.47] | * | 13 |

In-Bed Interval Evaluation (CCC and CCCLON) for Scripps Clinic and TCN

| Metric | Measure | Scripps Clinic | TCN | Difference | Difference Excludes 0 | Incomplete Bootstrap Samples |
| --- | --- | --- | --- | --- | --- | --- |
| SE | CCC | 0.34 [0.23, 0.45] | **0.50 [0.38, 0.59]** | -0.16 [-0.30, -0.04] | * |  |
| SOL | CCC | 0.19 [0.12, 0.26] | **0.84 [0.79, 0.88]** | -0.64 [-0.83, -0.41] | * |  |
| WASO | CCC | 0.38 [0.27, 0.48] | 0.46 [0.36, 0.55] | -0.08 [-0.23, 0.07] |  |  |
| TST | CCC | 0.90 [0.87, 0.92] | 0.91 [0.88, 0.93] | -0.01 [-0.03, 0.01] |  |  |
| SE | CCCLON | 0.31 [0.11, 0.48] | 0.41 [0.22, 0.57] | -0.10 [-0.28, 0.03] |  | 891 |
| SOL | CCCLON | 0.21 [0.05, 0.36] | **0.82 [0.75, 0.87]** | -0.61 [-0.90, -0.32] | * | 992 |
| WASO | CCCLON | 0.32 [0.14, 0.48] | 0.29 [0.13, 0.44] | 0.03 [-0.18, 0.26] |  | 2,017 |
| TST | CCCLON | 0.77 [0.66, 0.85] | 0.76 [0.65, 0.84] | 0.01 [-0.06, 0.09] |  | 0 |

**Notes:**

TCN = Temporal convolutional network

CCC = concordance correlation coefficient, calculated across days without respect to clustering within participants

CCCLON = concordance correlation coefficient with longitudinal repeated measures, calculated across days while accounting for clustering of days within participants

SE = Sleep efficiency

SOL = Sleep onset latency

WASO = Wake after sleep onset

TST = Total sleep time

Bolded values indicate the more favorable outcomes for comparisons with a 95% confidence interval of the difference excluding 0.

The CCCLON internally fits a mixed-effects model to the data to obtain variance components. Some permutations of the bootstrap can produce data sets that can’t be appropriately fit with a mixed-effects model (for example non-negative approximate variance-covariance). The column Incomplete Bootstrap Samples records the number of instances in which this occurred (out of 10,000).

The EcoSleep dataset was excluded from comparisons of SOL or SE, as the method of imputing in-bed labels for the EcoSleep data cannot identify pre-sleep wakefulness within the in-bed interval.

Supplemental References:

1. Boschloo RD. Raised conditional level of significance for the 2 × 2-table when testing the equality of two probabilities. *Statistica Neerlandica*. 1970;24(1):1-9. doi:10.1111/j.1467-9574.1970.tb00104.x

2. Virtanen P, Gommers R, Oliphant TE, et al. SciPy 1.0: Fundamental Algorithms for Scientific Computing in Python. *Nature Methods*. 2020;17:261-272. doi:10.1038/s41592-019-0686-2

3. Marino M, Li Y, Rueschman MN, et al. Measuring sleep: accuracy, sensitivity, and specificity of wrist actigraphy compared to polysomnography. *Sleep*. 2013;36(11):1747-1755. doi:10.5665/sleep.3142

4. Pedregosa F, Varoquaux G, Gramfort A, et al. Scikit-learn: machine learning in python. *Journal of Machine Learning Research*. 2011;12:2825−2830.

5. Tharwat A. Classification assessment methods. *Applied Computing and Informatics*. 2020;17(1):168-192. doi:10.1016/j.aci.2018.08.003

6. Byrt T, Bishop J, Carlin JB. Bias, prevalence and kappa. *Journal of Clinical Epidemiology*. 1993;46(5):423-429. doi:10.1016/0895-4356(93)90018-V

7. Lin LIK. A Concordance Correlation Coefficient to Evaluate Reproducibility. *Biometrics*. 1989;45(1):255-268. doi:10.2307/2532051

8. Menghini L, Cellini N, Goldstone A, Baker FC, de Zambotti M. A standardized framework for testing the performance of sleep-tracking technology: step-by-step guidelines and open-source code. *Sleep*. 2021;44(zsaa170). doi:10.1093/sleep/zsaa170

9. Sadeh A, Sharkey M, Carskadon MA. Activity-Based Sleep-Wake Identification: An Empirical Test of Methodological Issues. *Sleep*. 1994;17(3):201-207. doi:10.1093/sleep/17.3.201

10. Kripke DF, Hahn EK, Grizas AP, et al. Wrist actigraphic scoring for sleep laboratory patients: algorithm development. *Journal of Sleep Research*. 2010;19(4):612-619. doi:10.1111/j.1365-2869.2010.00835.x

11. Hammad G, Reyt M, Beliy N, et al. pyActigraphy: Open-source python package for actigraphy data visualization and analysis. *PLOS Computational Biology*. 2021;17(10):e1009514. doi:10.1371/journal.pcbi.1009514

12. Cole RJ, Kripke DF, Gruen W, Mullaney DJ, Gillin JC. Automatic sleep/wake identification from wrist activity. *Sleep*. 1992;15(5):461-469. doi:10.1093/sleep/15.5.461

13. Fawcett T. An introduction to ROC analysis. *Pattern Recognition Letters*. 2006;27(8):861-874. doi:10.1016/j.patrec.2005.10.010
